# Targeting Ash1L-STING Axis Restores NK Cell Function and Ameliorates Immune-Mediated Bone Marrow Failure Diseases

**DOI:** 10.7150/ijbs.131763

**Published:** 2026-07-13

**Authors:** Nan Song, Yi Cui, Li He, Xinrui Zhang, Yiyu Guo, Xinru Wu, Yu Gao, Chunyan Liu, Rong Fu

**Affiliations:** 1 Department of Hematology, Tianjin Medical University General Hospital, 154 Anshan Street, Heping District, Tianjin 300052, China, Tianjin Key Laboratory of Bone Marrow Failure and Malignant Hemopoietic Clone Control, Tianjin Institute of Hematology, State Key Laboratory of Experimental Hematology, China.; 2 The Third People's Hospital of Chengdu, Chengdu, China.

**Keywords:** aplastic anemia, NK cell, Ash1L, DNA damage repair, andrographolide

## Abstract

Aplastic anemia (AA) is an immune-mediated bone marrow failure (BMF) syndrome characterized by pancytopenia and bone marrow hypocellularity. While natural killer (NK) cell dysfunction contributes to AA pathogenesis, the epigenetic mechanisms linking genomic instability to inflammatory hyperactivation remain poorly defined. Here, we identify the histone methyltransferase Ash1L as a critical regulator of NK cell homeostasis in AA. Ash1L expression was markedly reduced in NK cells from AA patients and correlated with disease severity and elevated proinflammatory cytokines. Ash1L knockdown reduced NK cell viability, induced apoptosis and G1 cell-cycle arrest, and enhanced secretion of IL-6 and TNF-α. Mechanistically, Ash1L deficiency resulted in reduced H3K4 and H3K36 methylation, impairing activation of the ATM-CHK2-p53 signaling pathway, resulting in persistent γH2AX foci accumulation and aberrant activation of the cGAS-STING signaling pathway. Treatment with the natural compound Andrographolide (Andro) suppressed STING signaling, enhanced DNA damage repair efficiency, and partially restored NK cell function. In an immune-mediated BMF mouse model, Andro attenuated inflammatory responses, restored Ash1L expression in NK cells, inhibited STING pathway activation, and improved hematopoiesis. Collectively, these findings suggest that Ash1L acts as an epigenetic safeguard of genomic stability and inflammatory restraint in NK cells and highlight Andro as a potential therapeutic agent for AA and related immune-mediated BMF disorders.

## Introduction

Aplastic anemia (AA) is an immune-mediated bone marrow failure syndrome characterized by pancytopenia and hypocellular marrow. Various factors, including drugs, toxins, viral infections, and physical irradiation, are believed to trigger aberrant immune responses in susceptible individuals, which drives excessive activation of cytotoxic CD8^+^ T cells that attack hematopoietic stem cells (HSPCs) and eventually induce bone marrow failure (BMF)^1^. Current first-line treatment relies on immunosuppressive therapy (IST) centered on anti-thymocyte globulin (ATG)/anti-lymphocyte globulin (ALG) combined with cyclosporine (CsA) and/or eltrombopag^2^. While the majority of patients achieve complete remission, a subset remains refractory to IST. Recent studies have highlighted the complex immunopathology of AA, involving multiple immune cell types and their interactions. In addition to CD8^+^ T cells, natural killer (NK) cells, dendritic cells (DCs), and bone marrow stromal cells all contribute to disease onset and progression^3^-^5^. Therefore, deeper insights into the pathogenesis of AA are essential for developing novel therapeutic strategies.

NK cells are innate immune effectors capable of lysing virus-infected or malignant cells without prior antigen sensitization. They mediate cytotoxicity via perforin and granzyme release, and induce target cell apoptosis through cytokines such as tumor necrosis factor-alpha (TNF-α) and interferon-gamma (IFN-γ)^6^, ^7^. They also regulate immune homeostasis by eliminating overactivated immune cells and suppressing autoreactive T cells, playing immunoregulatory roles in autoimmunity^8^, ^9^. In AA, NK cells are numerically reduced and functionally impaired, compromising immune surveillance and the clearance of hyperactive autoreactive CD8^+^ T cells^10^. Single-cell sequencing and co-culture experiments have revealed profound dysregulation of NK cells and NK-like CD8^+^ T cells in the AA bone marrow. Although NK cells alone do not directly kill HSPCs, their secreted interferons enhance HSPC susceptibility to CD8^+^ T cell-mediated killing^11^. These findings indicate that NK cells are not merely effector killers but participate in AA pathogenesis through intricate immunoregulatory mechanisms. Elucidating the dysregulation of NK cell function is thus of significant importance for understanding the immunopathology of AA and developing new therapeutic approaches.

The histone methyltransferase Ash1L (absent, small, or homeotic discs 1-like; official gene symbol KMT2H), a conserved Trithorax group protein, catalyzes histone H3 lysine 36 (H3K36) methylation and counteracts Polycomb-mediated gene repression, thereby indirectly promoting H3K4 methylation and transcriptional activation^12^-^14^. Previous studies have demonstrated that Ash1L facilitates HOX gene transcription by counteracting Polycomb repressive complexes, and is implicated in development and neurodevelopmental disorders^15^, ^16^. In recent years, the role of Ash1L in immune regulation has attracted increasing attention. Elevated Ash1L expression suppresses proinflammatory gene transcription, attenuates Toll-like receptor signaling, reduces IL-6 and TNF production, and promotes regulatory T-cell differentiation, exerting protective effects in autoimmune disease models^17^-^19^. Given that AA is a bone marrow failure syndrome driven primarily by immune microenvironmental dysregulation^20^, whether Ash1L contributes to AA pathogenesis and the underlying mechanisms remains largely unexplored.

Natural products offer promising therapeutic leads. Andrographolide (Andro), a diterpenoid lactone from *Andrographis paniculata*, possesses anti-inflammatory and immunoregulatory properties^21^. Mechanistic studies have demonstrated that Andro directly inhibits the binding of NF-κB to DNA in multiple immune cells^22^, ^23^, thereby reducing the production of proinflammatory cytokines and chemokines^24^, although its precise mechanism in NK cells requires further validation. In addition, Andro can directly bind to dynamin-related protein 1 (DRP1), suppress excessive mitochondrial fission, conferring neuroprotective effects in neurodegenerative disease models^25^. Recent studies further demonstrate that Andro downregulates cGAS-STING pathway activation and promotes DNA damage repair (DDR) in intestinal epithelial cells, alleviating CPT-11-induced inflammatory mucosal injury^26^. Furthermore, the development of a novel Andro drug delivery system (HCL-PLGA-Ab) has highlighted its therapeutic potential in respiratory diseases^27^. Currently, multiple Andro-based formulations, including dripping pills, tablets, and injectable preparations, have been approved for clinical use in the treatment of viral infections, bacterial pneumonia, and cancer^28^, ^29^, underscoring its favorable druggability and translational potential. Given its pronounced anti-inflammatory and immunoregulatory effects, we hypothesized that Andro may also exert therapeutic benefits in immune-mediated BMF diseases, including AA.

In this study, we identify Ash1L as a critical regulator of DDR and inflammatory signaling in NK cells. We demonstrate that Ash1L downregulation in AA impairs DNA repair capacity, induces persistent DNA damage and aberrant STING activation, thereby amplifying inflammatory responses and disease severity. Importantly, Andro treatment restores DNA repair efficiency, suppresses STING signaling, partially rescues Ash1L expression, and improves immune dysregulation and hematopoietic failure, providing a potential therapeutic strategy for AA and related immune-mediated BMF diseases.

## Materials and Methods

### Study participants

This study enrolled 57 patients with AA who were treated at the Department of Hematology, Tianjin Medical University General Hospital from August 2021 to December 2023, including 20 newly diagnosed, 17 complete remission (CR), and 20 partial remission (PR) cases. Thirty age- and sex-matched healthy volunteers and patients with simple nutritional iron deficiency anemia were enrolled as control subjects. Diagnosis followed the Chinese Guidelines for the Diagnosis and Treatment of Aplastic Anemia (2022 edition)^30^, requiring: (1) at least two of the following: hemoglobin <100 g/L, platelets <50 × 10⁹/L, absolute neutrophil count <1.5 × 10⁹/L; (2) hypocellular or severely hypocellular bone marrow on multiple biopsies; (3) bone marrow biopsy showing reduced hematopoiesis, increased non-hematopoietic tissue, no reticulin fibrosis, and no abnormal cells; and (4) exclusion of congenital AA and other acquired or secondary bone marrow failure disorders. Complete remission was defined as hemoglobin >100 g/L, absolute neutrophil count >1.5 × 10⁹/L, and platelets >100 × 10⁹/L. The study was conducted according to the Declaration of Helsinki and approved by the Ethics Committee of Tianjin Medical University General Hospital. Written informed consent was obtained from all participants. Baseline characteristics are provided in Supplementary File 1.

### Antibodies and reagents

Antibodies against CD3-APC (340440) and CD56-PE (CYT-56PE-R) were obtained from BD Biosciences. Flow cytometry antibodies including Annexin V-FITC (556547), NKG2D-BV421 (320821), perforin-FITC (308103), and Granzyme B-BV421 (396413) were purchased from BD Biosciences. Antibodies against STING (80165-1-RR), pIRF3 (29528-1-AP), IRF3 (11312-1-AP), Vinculin (26520-1-AP), Tubulin (11224-1-AP), γH2AX (83307-2-RR) and H2AX (68888-1-Ig), ATM (27156-1-AP), CHK2 (13954-1-AP), P53 (10442-1-AP), ATR (19787-1-AP), CHK1 (25887-1-AP), RPA32 (10412-1-AP) were from Proteintech. Antibodies against TBK1 (S-658-50), pTBK1 (S-2481-14) were from STARETER. Antibodies against Ash1L (bs-11866R) were from Bioss. Antibodies against RBBP4 (EPR3411), RBBP7 (EPR23796-74), H3K36me2 (ab9049), H3K4me3 (ab8580) were from Abcam. Antibodies against pSTING (19781S), pATM (Ser1981) (13050), pCHK2 (Thr68) (2197), pP53 (Ser315) (82530), pATR (Ser428) (2853), pCHK1 (Ser345) (2348), pRPA32(S33) (10148) were from Cell Signaling Technology. NK cell isolation beads (130-092-661) were from Miltenyi Biotec. CD8⁺ T cell isolation was performed using CynScript kits (cat. no. L00684). Protease inhibitor cocktail (04693116001) was from Roche.

### Plasmids

The FLAG-tagged Ash1L C-terminal (Ash1L-C) construct was generated by RT-qPCR amplification and cloned into the PLV-3 × FLAG vector.

### Cell culture and NK cell isolation

Peripheral blood mononuclear cells (PBMCs) were isolated and resuspended in PBS containing 0.5% FBS and 2 mM EDTA. Non-NK cells were labeled with a biotin-conjugated antibody cocktail (Miltenyi Biotec) and removed using streptavidin-coated magnetic beads. The enriched NK cells (CD56⁺CD3⁻) were collected, washed, counted, and assessed for purity (>90%) by flow cytometry. Purified NK cells were cultured in complete RPMI-1640 medium (Gibco, 11875119) supplemented with 15% fetal bovine serum (FBS; Gibco, A5670701) and recombinant human cytokines, including IL-2 (500 U/mL; Procell, PB180634), IL-15 (20 ng/mL; Procell, PCK049), and IL-12 (5 ng/mL; Procell, PCK171). IL-2 and IL-15 were added at the time of seeding and replenished at the indicated concentrations every 2-3 days during medium replacement. IL-12 was supplied only during the initial 3 days of culture and subsequently reduced or withdrawn according to cell status.

### Flow cytometry

Cells were stained with CD56-PE (BD Pharmingen, cat. no. CYT-56PE-R) and CD3-APC (BD Pharmingen, cat. no. 345767). Prior to drug treatment, cells were seeded at a density of 1×10⁶ cells/mL. Cells were harvested, washed in cold PBS, and analyzed on a CytoFLEX flow cytometer (Beckman Coulter) using CytExpert software.

### Intracellular Cytokine Staining (ICS)

Intracellular cytokine staining (ICS): NK-92 cells were stimulated with PMA (50 ng/mL) and ionomycin (1 μM) for 4 hours in the presence of brefeldin A (10 μg/mL) to block cytokine secretion. Cells were then stained with CD56 antibody, fixed, permeabilized, and stained with antibodies against IFN-γ, IL-6, and TNF-α, followed by flow cytometric analysis. Three biological replicates were set up for each group to guarantee experimental reproducibility.

### Real-time RT-PCR

Total RNA was extracted using TRIzol reagent (Invitrogen) and reverse-transcribed with the Roche Reverse Transcription System. Quantitative PCR was performed using Power SYBR Green PCR Master Mix and a LightCycler 480 system (Roche), with β-actin as internal control. Primer sequences and Ash1L shRNA sequences are provided in Supplementary File 2.

### Immunopurification and silver staining

FLAG-Ash1L-C stable 293T cells were lysed in buffer containing protease inhibitors. Lysates from ~ 6 × 10⁹ cells were incubated with anti-FLAG M2 affinity resin (1 mL), washed with PBS containing 0.2% NP-40, and eluted with FLAG peptide. Eluates were resolved on NuPAGE 4-12% Bis-Tris gels, silver-stained (Pierce), and bands subjected to LC-MS/MS analysis (Supplementary File 3).

### Immunoprecipitation

Cells were lysed in NETN buffer (50 mM Tris-HCl pH 8.0, 150 mM NaCl, 0.2% NP-40, 2 mM EDTA) containing protease inhibitors at 4°C for 30 min. Clarified lysates (500 μg protein) were incubated with control IgG or specific antibodies (1-2 μg) overnight at 4°C, followed by 2 h with protein G magnetic beads (Invitrogen). Beads were washed five times, and bound proteins were eluted by boiling in 2× SDS loading buffer, separated by SDS-PAGE, and analyzed by Western blotting.

### Nucleosome digestion assay

NK-92 cells were washed in PBS, lysed in MN buffer (50 mM KCl, 8 mM MgCl₂, 2 mM CaCl₂, 100 mM Tris-HCl) containing protease inhibitors at 4°C, and nuclei isolated by centrifugation. Nuclei were resuspended in glycerol buffer and digested with micrococcal nuclease (various concentrations) in the presence of CaCl₂ at 37°C for 5 min. Reactions were stopped with EDTA, followed by RNase A and proteinase K treatment. Purified DNA was resolved on 1.2% agarose gels for nucleosome mapping.

### Comet assay

Performed according to the manufacturer's protocol with minor modifications. Cells (1×10⁶/mL) were embedded in a dual-layer agarose system, lysed in cold solution with 10% DMSO at 4°C, subjected to alkaline electrophoresis, neutralized, stained with PI or DAPI, and imaged using fluorescence microscopy.

### Immunofluorescence

Cells grown on coverslips were fixed in 2% paraformaldehyde, permeabilized with 0.2% Triton X-100, blocked with 5% donkey serum, incubated with primary antibodies, and stained with Alexa Fluor 488/594 secondary antibodies. Confocal images were acquired using an Olympus FluoView 1000 microscope with a ×60 oil-immersion lens.

### X-ray irradiation and laser microirradiation

Cells grown in glass-bottom dishes were transfected with GFP-Ash1L-C. Irradiation was performed using an RS2000 PRO X-ray generator (Radsource; 160 kV, 25 mA) or pulsed nitrogen laser (365 nm, 16 Hz, 50% output) coupled to a Leica confocal system to induce single- and double-strand breaks. Signal intensity along irradiation paths was quantified using ImageJ.

### Western blotting

Whole-cell lysates were prepared, resolved in 5×SDS-PAGE loading buffer, boiled, separated by SDS-PAGE, and probed with primary and secondary antibodies.

### Cell Counting Kit-8

Cell viability was assessed using the Cell Counting Kit-8 (CCK-8) assay (Beyotime Biotechnology, China) according to the manufacturer's instructions. Briefly, cells were collected and counted, then seeded into 96-well plates at a density of 1×10⁴ cells per well in a final volume of 100 μL. Subsequently, 10 μL of CCK-8 solution was gently added to each well, avoiding the formation of bubbles, as bubbles may interfere with absorbance readings. To minimize liquid evaporation during incubation, the peripheral wells of the 96-well plate were filled with PBS or culture medium. The plates were then incubated in a light-protected cell culture incubator at 37°C for 2 hours. After incubation, absorbance was measured at 450 nm using a microplate reader (Thermo Fisher Scientific, USA). Cell viability was calculated using the following formula: Cell viability (%) = [(As-Ab) / (Ac-Ab)]×100%, where As represents the absorbance of the experimental wells, Ac represents the absorbance of the control wells, and Ab represents the absorbance of the blank wells.

### Immune-mediated BMF mouse model and administration methods

B6D2F1 recipient mice received sublethal total body irradiation (4.5 Gy), followed by adoptive transfer of 5 × 10⁷ splenic lymphocytes isolated from C57BL/6 donor mice within 4-6 h post-irradiation. Vehicle control group received equal-volume PBS; treatment groups were administered Andro (10 mg/kg), CsA (5 mg/kg), or combined CsA + Andro every two days for 14 consecutive days. Mice were monitored daily and sacrificed on day 15 for peripheral blood analysis and tissue collection.

### Tissue specimens

Sternum samples were fixed in 10% formalin and processed for hematoxylin and eosin staining. Images were acquired at multiple magnifications and analyzed using Image-Pro Plus for quantitative assessment.

### Statistical analysis

Data were presented as mean ± SD and analyzed with GraphPad Prism 8. Normality was verified by Shapiro-Wilk test. Comparisons between two/multiple groups adopted parametric or nonparametric tests accordingly. Pearson correlation was applied for linear relationships. Two-tailed tests were used, with *P* < 0.05 considered significant.

## Results

### Reduced Ash1L expression in NK cells from AA patients impairs NK cell function

**Figure 1:**
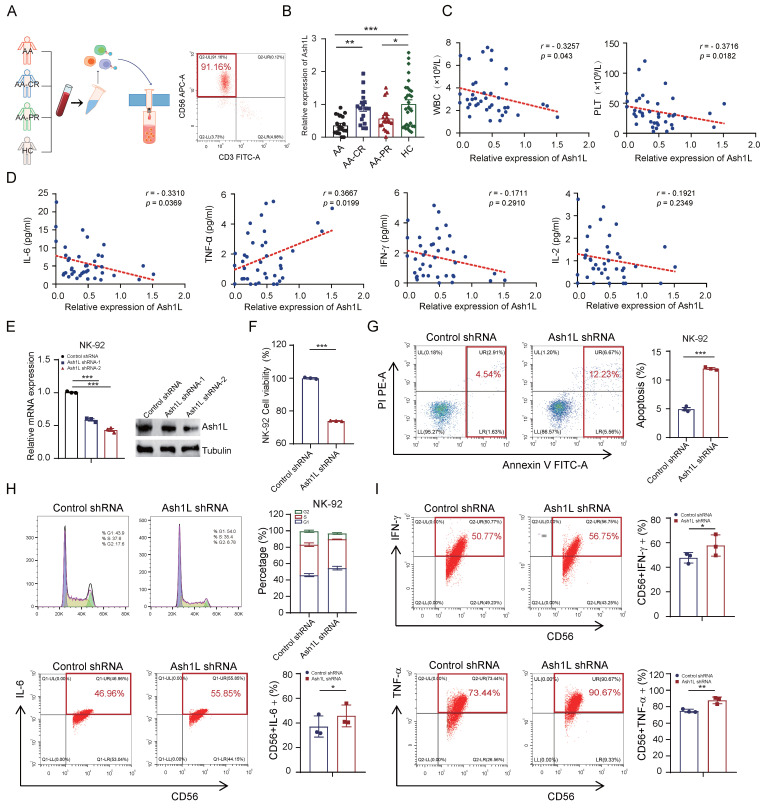
** Reduced Ash1L expression impairs NK cell function in AA.** (A) Bone marrow NK cells were isolated from patients with AA, partial remission (AA-PR), complete remission (AA-CR), and healthy controls (HC). Flow cytometry (FCM) was performed to detect the purity of isolated bone marrow NK cells. (B) Relative Ash1L mRNA expression in isolated bone marrow NK cells was measured by RT-qPCR. (C) Correlation analysis between Ash1L expression in isolated bone marrow NK cells and peripheral blood parameters in AA and AA-PR patients. (D) Correlation analysis between Ash1L expression of bone marrow NK cells and serum cytokine levels in AA and AA-PR patients. (E) Knockdown efficiency of Ash1L in NK-92 cells assessed by qPCR and western blot (WB). (F) Cell viability of NK-92 cells following Ash1L knockdown assessed by CCK-8 assay. (G) Apoptosis of NK-92 cells analyzed by FCM after Ash1L knockdown. (H) Cell cycle distribution of NK-92 cells following Ash1L knockdown. (I) Cytokine production by NK-92 cells after Ash1L knockdown assessed by FCM. *p < 0.05, **p < 0.01, ***p < 0.001, one-way ANOVA.

Ash1L is a histone methyltransferase that catalyzes H3K36 methylation, promoting transcriptional activation and counteracting Polycomb-mediated gene repression. It has been implicated in the regulation of developmental genes, DNA damage repair, and modulation of inflammatory responses in various immune cell types. To investigate the potential role of Ash1L in aplastic anemia (AA), we enrolled a total of 57 AA patients, including 20 treatment-naïve patients (primary untreated group), 17 patients in complete remission (CR), and 20 patients in partial remission (PR), along with 30 healthy controls (HC). Bone marrow mononuclear cells were isolated, and NK cells were purified using magnetic bead-based negative selection. Flow cytometric (FCM) analysis confirmed an NK cell purity (CD3^-^CD56^+^) exceeding 90% (Figure 1A). Quantitative real-time PCR (RT-qPCR) analysis revealed that Ash1L mRNA expression was significantly reduced in NK cells from the untreated AA and PR groups compared with the HC group, whereas Ash1L expression in the CR group was markedly elevated relative to the untreated AA group (Figure 1B).

Correlation analysis in the untreated AA and PR groups showed that Ash1L expression levels were negatively correlated with white blood cell (WBC) count, platelet (PLT) count, reticulocyte (Ret) percentage, and absolute Ret count, but not with red blood cell (RBC) count or hemoglobin (Hb) levels (Figure 1C and Figure S1A). Previous studies have reported dysregulated serum cytokine levels, including TNF-α, IFN-γ, IL-2, and IL-6, in AA patients. Therefore, serum cytokine levels were measured by flow cytometry. Although the mean levels of IL-6, IL-10, TNF-α, IFN-γ, and IL-17A were elevated in the AA and AA-PR groups compared with healthy controls, only IL-6 in the AA group, IL-4 and TNF-α in the AA-PR group, and IL-17A in the AA-CR group reached statistical significance (Figure S1B). Further correlation analyses revealed that Ash1L expression was negatively correlated with IL-6 levels and positively correlated with TNF-α levels, while no significant correlations were observed with other cytokines (Figure 1D and Figure S1C).

To investigate the functional role of Ash1L, we transduced NK-92 cells with lentiviral shRNAs targeting Ash1L. RT-qPCR and Western blot (WB) analyses confirmed knockdown efficiency, with the Ash1L-2 shRNA exhibiting superior silencing (Figure 1E). This shAsh1L-2 construct was selected for subsequent experiments and similarly effectively reduced Ash1L expression in the YT NK cell line (Figure S1D). Cell viability assays (CCK-8) assays revealed reduced cell viability in Ash1L-knockdown NK-92 and YT cells compared with controls (Figure 1F and Figure S1E). FCM analysis indicated significantly increased apoptosis in Ash1L-knockdown NK-92 and YT cells (Figure 1G and Figure S1F). Cell cycle analysis showed that Ash1L knockdown led to G1 cell-cycle arrest, with increased G1 phase proportion, decreased G2 phase, and no significant change in S phase in both NK-92 and YT cells (Figure 1H and Figure S1G). Further analyses revealed that Ash1L knockdown increases p21 mRNA levels while decreasing Cyclin D1 mRNA in NK-92 cells, confirming that Ash1L deficiency induces G1 phase cell cycle arrest (Figure S1H). Finally, FCM analysis demonstrated that Ash1L knockdown led to increased expression of IFN-γ, IL-6, and TNF-α in NK-92 cells to varying degrees (Figure 1I). These findings indicate that Ash1L expression is reduced in NK cells from AA patients and that its downregulation impairs NK cell viability, promotes apoptosis and cell cycle arrest, and alters cytokine production, thereby influencing NK cell function.

### Ash1L regulates DDR

**Figure 2:**
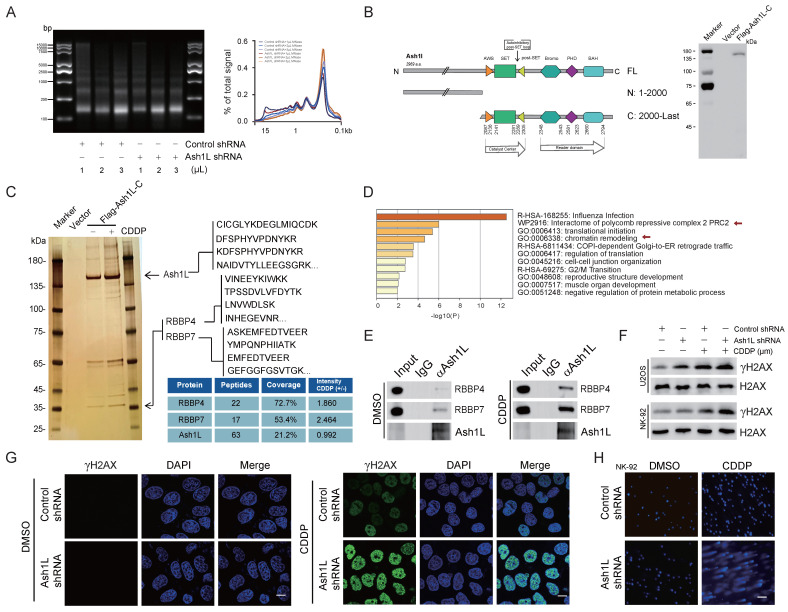
** Ash1L regulates DNA damage repair.** (A) Nucleosome digestion assay showing chromatin accessibility in NK-92 cells after Ash1L knockdown. (B) Construction and validation of the Ash1L C-terminal truncation (Ash1L-C). (C) Silver staining-based mass spectrometry analysis of Ash1L-interacting proteins with or without cisplatin (CDDP) treatment in U2OS cells. (D) KEGG pathway enrichment analysis of differentially enriched proteins (unique peptides ≥5, coverage ≥15%, fold change ≥1.5). (E) Endogenous Co-IP showing interactions between Ash1L and RBBP4/RBBP7 with or without CDDP in NK-92 cells. (F) γH2AX expression in U2OS and NK-92 cells with or without Ash1L knockdown under CDDP treatment. (G) Immunofluorescence (IF) analysis of γH2AX foci in U2OS cells following Ash1L knockdown. Scale bar, 10 μm. (H) Comet assay assessing DNA damage in NK-92 cells after Ash1L knockdown. Scale bar, 100 μm.

Ash1L catalyzes H3K36me2, antagonizes PRC2-mediated gene silencing and contributes to the maintenance of genomic stability^16^. Nucleosome digestion assays revealed that Ash1L knockdown in NK-92 and YT cells resulted in reduced nucleosome cleavage efficiency, indicating chromatin compaction and decreased chromatin accessibility (Figure 2A and Figure S2A). Reduced chromatin accessibility may impede the recruitment of DDR proteins to chromatin, leading to defective DDR. Given that Ash1L has previously been reported to participate in nucleotide excision repair (NER)^31^, we sought to determine whether Ash1L influences DDR in the context of AA. To address this, we treated NK-92 cells with varying concentrations of the DNA cross-linker cisplatin (CDDP)^32^. Phosphorylation of histone H2AX at Ser139 (γH2AX) is a canonical biomarker of DNA damage^33^. We found that 40 μM CDDP significantly increased DNA damage in NK-92 cells (Figure S2B). Further analysis showed that exposure to 40 μM CDDP for 8 h induced robust DNA damage in both NK-92 cells and U2OS cells, a commonly used model for DNA damage studies (Figure S2C). Therefore, this condition was selected for subsequent experiments.

To identify potential Ash1L-interacting proteins, silver staining-based mass spectrometry was performed. Because full-length Ash1L (>340 kDa) is expressed at relatively low levels and is difficult to overexpress, we analyzed its domain structure and cloned the C-terminal region (amino acids 2000-2969), which contains the functional SET domain, into an expression vector. A stable U2OS cell line expressing FLAG-tagged Ash1L C-terminal fragment (FLAG-Ash1L-C) was established, and successful expression was confirmed (Figure 2B). Silver staining-mass spectrometry was then conducted in FLAG-Ash1L-C cells in the presence or absence of CDDP (40 μM, 8 h) (Figure 2C). Proteins with unique peptides ≥5, coverage ≥15%, and a fold change ≥1.5 between the CDDP-treated and untreated groups were subjected to KEGG pathway enrichment analysis, which revealed significant enrichment in pathways related to influenza infection, PRC2-associated protein interaction networks, and chromatin remodeling (Figure 2D). Given the critical role of PRC2 in chromatin organization and DDR^34^, and the known antagonistic relationship between Ash1L and PRC2, these results support the reliability of the proteomic analysis. Among the differentially enriched proteins, RBBP4 and RBBP7, both of which are known to participate in chromatin remodeling and DNA repair^35^, were prominently enriched and exhibited high confidence scores under DNA damage conditions (Figure 2C). Endogenous co-immunoprecipitation (Co-IP) assays in NK-92 cells further confirmed that Ash1L interacts with RBBP4 and RBBP7, and that these interactions were markedly enhanced upon CDDP treatment (Figure 2E).

Ash1L knockdown resulted in a significant increase in γH2AX protein levels in both NK-92 and U2OS cells following CDDP treatment (Figure 2F). Similarly, upon X-ray irradiation, γH2AX expression was markedly elevated in Ash1L-deficient cells compared with controls in NK-92 cells (Figure S2D). Similarly, the immunofluorescence (IF) analysis revealed substantially higher γH2AX foci in Ash1L-knockdown cells compared with controls under both CDDP-induced chemical damage and X-ray-induced physical damage in U2OS cells (Figure S2E, Figure 2G and Figure S2F). Furthermore, comet assays revealed that Ash1L knockdown led to more pronounced DNA tail formation in both NK-92 and U2OS cells, indicating increased DNA damage accumulation (Figure 2H, Figure S2G, and Figure S2H). Collectively, these results demonstrate that Ash1L deficiency aggravates DNA damage and establish Ash1L as an important positive regulator of DDR.

### Ash1L participates in DDR through its enzymatic activity

**Figure 3:**
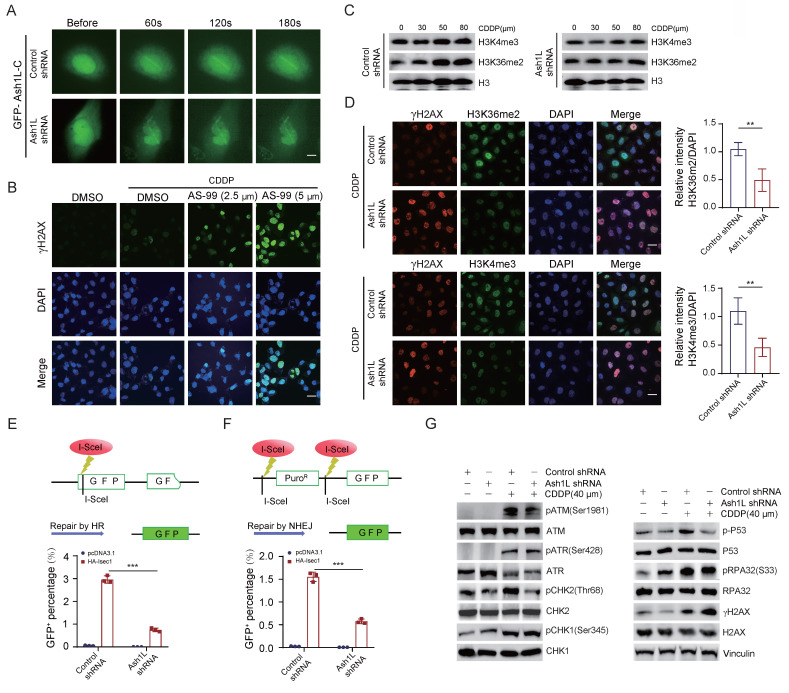
** Ash1L promotes DNA damage repair via its enzymatic activity.** (A) Recruitment of GFP-Ash1L-C to laser-induced DNA double-strand breaks (DSBs) in control and Ash1L-knockdown U2OS cells. Scale bar, 5 μm. (B) γH2AX fluorescence intensity in cells treated with increasing concentrations of the Ash1L enzymatic inhibitor AS-99 in U2OS cells. Scale bar, 25 μm. (C) WB analysis of H3K36me2 and H3K4me3 levels in control and Ash1L-knockdown cells treated with increasing concentrations of CDDP in NK-92 cells. (D) IF analysis of H3K36me2, H3K4me3, and γH2AX following Ash1L knockdown in U2OS cells. Scale bar, 25 μm. (E) Homologous recombination (HR) reporter assay after Ash1L knockdown in U2OS cells. (F) Non-homologous end joining (NHEJ) reporter assay after Ash1L knockdown in U2OS cells. (G) WB analysis of ATM/ATR pathway activation in control and Ash1L-knockdown cells in NK-92 cells. **p < 0.01, ***p < 0.001, one-way ANOVA.

To determine whether Ash1L directly participates in DDR, we performed laser microirradiation assays. GFP-Ash1L-C, which contains the enzymatic SET domain, was robustly recruited to laser-induced double-strand breaks (DSBs). In contrast, recruitment was markedly attenuated upon Ash1L knockdown in U2OS cells (Figure 3A and Figure S3A). Given that GFP-Ash1L-C contains the catalytic domain of Ash1L, we further investigated whether its role in promoting DDR depends on its methyltransferase activity. Treatment with the Ash1L enzymatic inhibitor AS-99 resulted in a dose-dependent increase in γH2AX fluorescence intensity in U2OS cells, indicating impaired DNA repair efficiency upon inhibition of Ash1L catalytic activity (Figure 3B and Figure S3B). H3K36me2 levels increase at DSB sites and facilitate non-homologous end joining (NHEJ) repair^36^, ^37^. As Ash1L is a direct histone methyltransferase for H3K36me2 and also antagonizes PRC2 to indirectly influence H3K4me3^14^, we next examined changes in these histone modifications following Ash1L knockdown. We found that depletion of Ash1L resulted in reduced global levels of both H3K4me3 and H3K36me2. Moreover, upon increasing concentrations of CDDP, H3K4me3 and particularly H3K36me2 levels gradually increased in control cells in response to DNA damage, whereas this damage-induced elevation of H3K36me2 and H3K4me3 was markedly attenuated in Ash1L-knockdown NK-92 cells (Figure 3C). IF staining corroborated these findings in U2OS cells, showing reduced fluorescence intensity for both marks upon Ash1L knockdown, with a more pronounced decrease in H3K36me2 (Figure 3D). Thus, while Ash1L promotes methylation of both H3K4 and H3K36, it primarily contributes to DDR through enhancement of H3K36me2.

Both X-ray irradiation and CDDP induce single- and double-strand breaks, which are predominantly repaired via homologous recombination (HR) and non-homologous end joining (NHEJ). HR repair is primarily mediated by activation of ATR, RPA, and CHK1, whereas NHEJ involves recognition of DNA ends by the Ku70/Ku80 heterodimer, recruitment of DNA-PK, and subsequent activation of ATM and its downstream effectors CHK2, p53, as well as recruitment of 53BP1^38^. Using HR and NHEJ reporter assays, we found that knockdown of Ash1L significantly reduced the repair efficiency of both HR and NHEJ pathways in U2OS cells (Figures 3E and Figure 3F). IF staining also showed that the fluorescence intensities of BRCA1 and 53BP1, were markedly decreased upon Ash1L knockdown in U2OS cells (Figure S3C). Given that ATM/ATR function as DNA damage sensors, CHK2/CHK1 as mediators, and p53 as an effector in DDR signaling^39^, we next examined the activation status of these key molecules. WB analysis revealed that phosphorylation levels of ATM (Ser1981), CHK2 (Thr68), and p53 (Ser315) were significantly decreased in Ash1L-depleted NK-92 cells compared with controls. In contrast, phosphorylation levels of ATR (Ser428), CHK1 (Ser345), and RPA32 (Ser33) were not significantly altered (Figure 3G). These findings suggest that Ash1L primarily facilitates DDR through activation of the ATM-CHK2-p53 signaling pathway.

### Ash1L deficiency impairs DDR efficiency in NK cells

**Figure 4:**
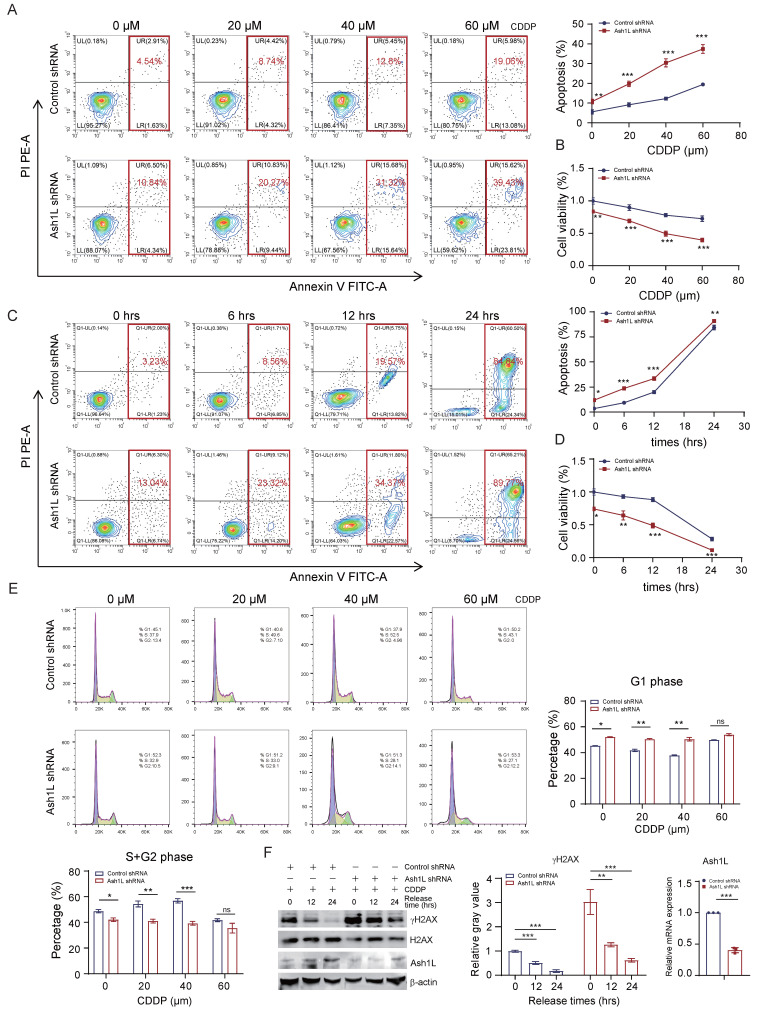
** Ash1L deficiency impairs DNA damage repair efficiency in NK cells.** (A) Apoptosis of control and Ash1L-knockdown NK-92 cells after 8 h treatment with increasing concentrations of CDDP. (B) Cell proliferation assessed by CCK-8 assay under the same conditions as (A). (C) Apoptosis analysis following 40 μM CDDP treatment for different durations in control and Ash1L-knockdown NK-92 cells. (D) Cell proliferation assessed under the same conditions as (C). (E) Cell cycle distribution of NK-92 cells treated with increasing concentrations of CDDP. (F) NK-92 cells were treated with 40 μM CDDP for 8 h, followed by recovery in fresh medium; γH2AX levels were assessed by Western blot at indicated time points. *p < 0.05, **p < 0.01, ***p < 0.001, ns, not significant, one-way ANOVA.

During DDR, cell cycle progression is halted to facilitate repair. For instance, p53, acting as an effector in DDR pathways, induces G1/M arrest; successful repair allows cycle resumption, whereas persistent unrepaired damage triggers programmed cell death or apoptosis^40^. Previously, we demonstrated that Ash1L knockdown increases apoptosis, reduces proliferation, and induces cell cycle arrest. We next examined the impact of reduced Ash1L expression on apoptosis, proliferation, and cell cycle in the context of DNA damage. Upon treatment with increasing concentrations of CDDP, Ash1L-depleted NK-92 cells exhibited a significant increase in total apoptosis compared with control cells (Figure 4A) and a marked reduction in proliferation (Figure 4B). Similarly, at a fixed CDDP concentration, prolonged exposure further exacerbated apoptosis and suppressed proliferation in Ash1L-knockdown NK-92 cells relative to controls (Figure 4C and Figure 4D). Cell cycle analysis revealed that, under DNA damage, Ash1L-deficient NK-92 cells remained predominantly arrested in G1 phase, consistent with previous observations. Interestingly, in control cells, increasing CDDP concentrations led to a gradual reduction of G1-phase cells and a corresponding increase in S+G2-phase cells, reflecting DNA damage-induced cell cycle remodeling. In contrast, Ash1L-depleted NK-92 cells showed no significant changes in G1 or S+G2 phase proportions with increasing CDDP doses (Figure 4E), indicating failure of cell cycle progression and remodeling due to impaired HR/NHEJ repair and defective ATM-CHK2-p53 signaling. Consequently, unresolved DNA damage drives cells toward apoptosis. Finally, NK-92 cells were treated with CDDP for 8 hours to induce damage, followed by replacement with fresh medium and continued culture. Cells were harvested at various time points post-recovery. Compared with controls, Ash1L-knockdown cells displayed persistent γH2AX expression, indicating sustained DNA damage (Figure 4F). These findings demonstrate that reduced Ash1L expression markedly slows DDR efficiency in NK cells, leading to prolonged damage persistence.

### Andro attenuates STING pathway activation induced by Ash1L deficiency

**Figure 5:**
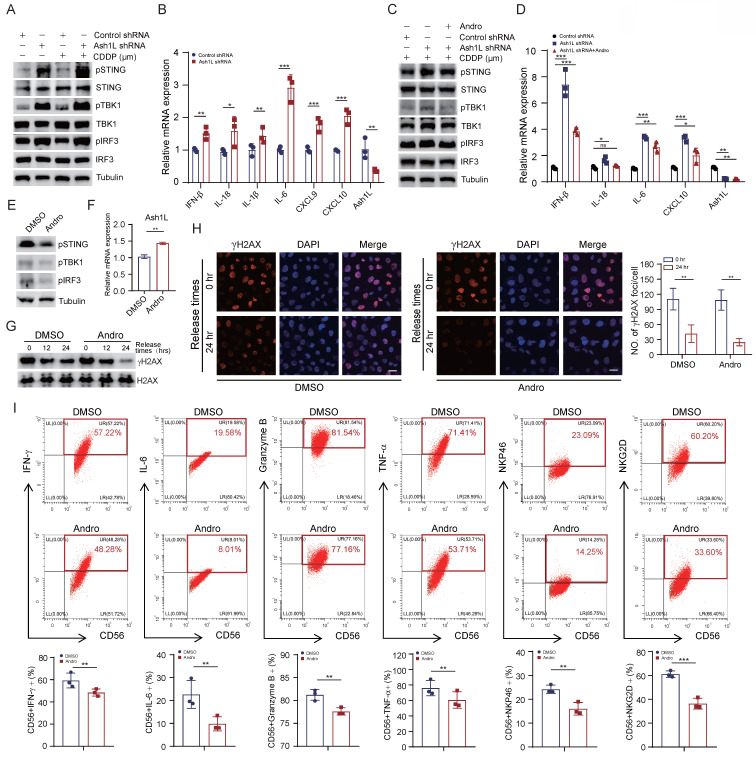
** Andrographolide attenuates STING pathway activation induced by Ash1L deficiency.** (A) WB analysis of STING pathway activation in NK-92 cells after Ash1L knockdown. (B) qPCR analysis of STING downstream gene expression following Ash1L knockdown in NK-92 cells. (C) STING pathway inhibition in Ash1L-knockdown NK-92 cells treated with Andrographolide (Andro). (D) qPCR analysis of STING downstream genes under the same conditions as (C). (E) STING pathway inhibition in NK-92 cells treated with Andro. (F) Ash1L mRNA expression in NK-92 cells following Andro treatment. (G) γH2AX expression during recovery from CDDP-induced DNA damage with or without Andro treatment in NK-92 cells. (H) IF analysis of γH2AX foci in U2OS cells under the same conditions as (G). (I) Cytokine production by NK-92 cells after Andro treatment. Scale bar, 25 μm. *p < 0.05, **p < 0.01, ***p < 0.001, ns, not significant, one-way ANOVA.

Previous studies have shown that defects in DSB repair lead to persistent DNA damage and leakage of damaged nuclear DNA into the cytoplasm^41^. Cytoplasmic DNA acts as a potent stimulus for innate immune activation. Consistently, we observed a significant increase in micronuclei formation in Ash1L-knockdown U2OS cells compared with controls (Figure S4A). Cytoplasmic DNA is sensed by the cGAS-STING pathway, a key mediator of both innate and adaptive immunity, which triggers downstream phosphorylation events, ultimately inducing the expression of interferon-stimulated genes (ISGs)^42^. In addition, Ash1L depletion in U2OS cells led to a marked increase in reactive oxygen species (ROS) levels (Figure S4B), which may further contribute to STING pathway activation. Further WB analysis revealed that knockdown of Ash1L significantly increased the phosphorylation levels of STING, TBK1, and IRF3, indicating activation of the STING signaling pathway in NK-92 cells (Figure 5A). Activation of the STING-TBK1-IRF3 axis promotes the production of proinflammatory cytokines, including IFN-β, IL-18, and CXCL10^43^. As expected, Ash1L deficiency led to elevated levels of type I IFN (IFN-β) and the chemokine CXCL10 (Figure 5B).

Andrographolide (Andro), a diterpenoid compound isolated from Andrographis paniculata, possesses anti-inflammatory, anti-microbial, and detoxifying properties^21^. To determine the appropriate working concentration of Andro for subsequent experiments, we first evaluated its inhibitory effect on NK-92 cells. The results showed that Andro suppressed cell viability in a dose-dependent manner, with an IC₅₀ value of 17.06 μM (Figure S4C). Based on the IC₅₀ results, we selected 20 μmol/L Andro for the following mechanistic studies. Treatment with Andro significantly reduced the Ash1L knockdown-induced phosphorylation of STING, TBK1, and IRF3, as well as the elevated expression of IFN-β, CXCL10, and IL-18 in NK-92 cells (Figure 5C and Figure 5D). Moreover, Andro alone was able to suppress basal STING signaling in NK-92 cells, consistent with previous reports^44^ (Figure 5E), and partially upregulated Ash1L mRNA expression (Figure 5F). Further research has revealed that Andro upregulates Ash1L mRNA and protein expression in a dose-dependent manner (Figure S4D). To assess whether Andro could enhance DNA repair, NK-92 cells were treated with CDDP for 8 hours and subsequently cultured in normal medium for different recovery periods. WB showed that γH2AX levels were markedly reduced in Andro treated cells compared with controls (Figure 5G). IF in U2OS cells similarly demonstrated attenuated γH2AX foci in Andro-treated cells 24 hours post-damage (Figure 5H). These results indicate that Andro enhances DDR efficiency. Finally, Andro treatment of NK-92 cells reduced the expression of IFN-γ, IL-6, and TNF-α, suggesting that Andro can partially suppress NK cell overactivation and limit proinflammatory responses (Figure 5I). These findings demonstrate that Andro attenuates STING pathway hyperactivation resulting from reduced Ash1L expression, promotes DDR, and suppresses overactivation of NK cells.

### STING pathway inhibition alleviates BMF *in vivo*

**Figure 6:**
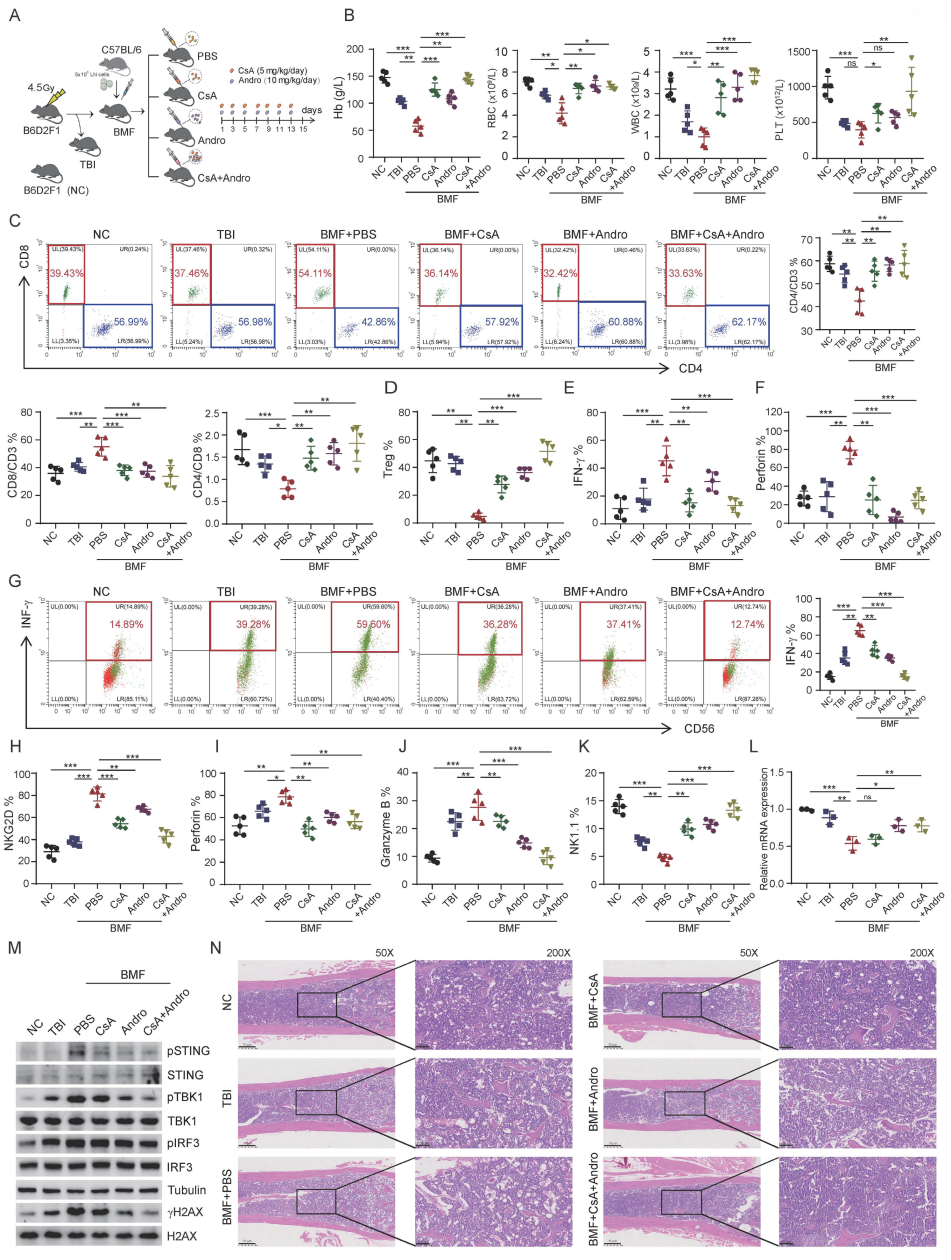
** Andro ameliorates immune-mediated BMF *in vivo*.** (A) Experimental design of the BMF mouse model and treatment regimen. (B) Peripheral blood counts (WBC, RBC, Hb, PLT) on day 15 post-induction. (C) FCM analysis of bone marrow T cell subsets. (D) Proportion of regulatory T cells (Tregs) in bone marrow. (E-F) IFN-γ and perforin expression in bone marrow CD8⁺ T cells. (G-J) IFN-γ, NKG2D, perforin and granzyme B expression in bone marrow NK cells. (K) Proportion of NK cells in bone marrow. (L) Ash1L mRNA expression in bone marrow NK cells detected by RT-qPCR. (M) WB analysis of DNA damage and STING pathway related proteins in NK cells. (N) Representative H&E staining of sternal bone marrow sections on day 15. *p < 0.05, **p < 0.01, ***p < 0.001, ns, not significant, one-way ANOVA.

To evaluate the therapeutic efficacy of Andro in immune-mediated BMF *in vivo*, BMF mouse models were established using C57BL/6 and B6D2F1 mice. BMF mice were randomly assigned to receive PBS (untreated), cyclosporine A (CsA), Andro, or combination treatment (CsA+Andro) (Figure 6A). On day 15 post-model induction, peripheral blood analysis revealed that WBC, RBC, Hb, and PLT counts were significantly higher in the CsA, Andro, and CsA+Andro groups compared with PBS treated BMF mice (Figure 6B). FCM analysis of bone marrow T cell subsets showed that BMF mice exhibited a reduction in CD4^+^/CD3^+^ T cells, an increase in CD8^+^/CD3^+^ T cells, and a decreased CD4^+^/CD8^+^ ratio relative to normal controls, consistent with the AA phenotype. Treatment with Andro or CsA+Andro restored the CD4^+^/CD3^+^ T cell proportion, reduced CD8^+^/CD3^+^ T cell frequency, and increased the CD4^+^/CD8^+^ ratio, along with an increase in Treg cell proportion (Figure 6C and Figure 6D), indicating that Andro, similar to CsA, can normalize T cell subset distribution in BMF mice. Moreover, Andro treatment significantly reduced IFN-γ and perforin expression in CD8^+^ T cells, suggesting alleviation of CD8^+^ T cell hyperactivation (Figure 6E and Figure 6F).

Next, we analyzed NK cell function in BMF mice. Compared with untreated BMF mice, NK cells from the CsA and Andro groups exhibited reduced IFN-γ expression, with combination treatment showing the most pronounced effect (Figure 6G). Similarly, NKG2D, perforin, and granzyme B levels were decreased to varying degrees following CsA or Andro treatment (Figures 6H-J), and the overall NK cell proportion increased post-treatment (Figure 6K), indicating functional restoration of NK cells *in vivo*. Sorted NK cells were subjected to RT-qPCR, which revealed that Andro treatment significantly restored Ash1L mRNA expression compared with CsA treatment (Figure 6L). WB analysis showed that Andro markedly reduced γH2AX expression in NK cells and inhibited activation of the STING signaling pathway(Figure 6M). These results suggest that, unlike CsA, Andro may exert its effects through targeted upregulation of Ash1L expression and suppression of the STING pathway in NK cells. Finally, histological examination of bone marrow revealed that untreated BMF mice displayed markedly reduced hematopoietic cellularity and numerous adipocytes, whereas CsA and Andro treatment significantly improved marrow cellularity (Figure 6N). Collectively, these data demonstrate that Andro inhibits CD8^+^ T cell cytotoxic function, restores NK cell activity, and suppresses the STING signaling pathway in NK cells, ultimately reducing proinflammatory cytokine levels in the bone marrow immune microenvironment and ameliorating BMF. Thus, Andro exhibits therapeutic potential for BMF syndromes to a certain extent.

## Discussion

**Figure 7:**
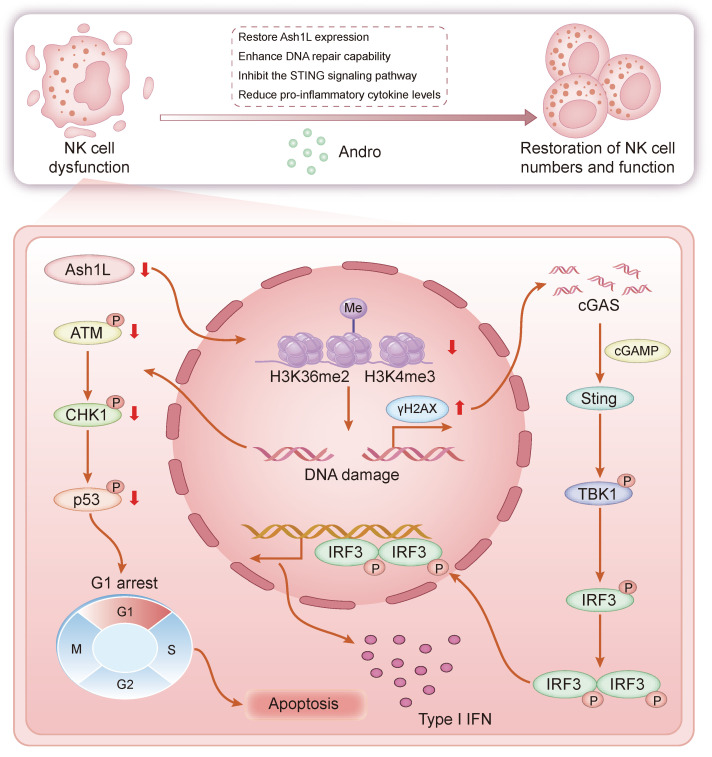
** Schematic model.** Schematic illustrating the proposed model of Ash1L downregulation in AA pathogenesis and therapeutic intervention by Andro.

In this study, we demonstrate that Ash1L expression is downregulated in bone marrow NK cells from patients with AA and that this downregulation promotes disease pathogenesis and progression. Reduced Ash1L expression leads to impaired NK cell proliferation, increased apoptosis, and cell cycle arrest. Mechanistically, Ash1L regulates H3K36me2 levels through its methyltransferase activity, thereby activating the ATM-CHK2-p53 signaling pathway and maintaining HR- and NHEJ-mediated DDR. Under conditions of endogenous or exogenous genotoxic stress, reduced Ash1L expression in NK cells markedly compromises DNA repair efficiency, leading to sustained γH2AX accumulation and aberrant activation of the cGAS-STING pathway, which in turn drives excessive inflammatory cytokine production and ultimately exacerbates bone marrow failure (Figure 7). Andro partially restores Ash1L expression, enhances DDR, suppresses STING signaling, reduces inflammatory cytokines, and rescues NK cell function both *in vitro* and *in vivo*, thereby ameliorating immune dysregulation and bone marrow failure. This study, for the first time, reveals the critical role of Ash1L in maintaining genomic stability and functional homeostasis in AA NK cells from an epigenetic modification perspective, and provides a solid theoretical foundation for the potential clinical application of Andro in AA and other immune-mediated BMF diseases.

Previous studies have shown that Ash1L silencing increases susceptibility to inflammatory osteoarthritis and autoimmune uveitis in mice^17^, ^45^. Ash1L also participates in the regulation of pro- and anti-inflammatory cytokines such as IL-10 and IL-6, and is closely associated with multiple autoimmune diseases^18^, ^46^. The present study further confirms that NK cells in AA, an autoimmune bone marrow failure syndrome, exhibit reduced Ash1L expression accompanied by functional impairment. Given that Ash1L plays important roles in macrophages and other innate lymphoid cells^19^, ^47^, we speculate that aberrant Ash1L expression may also occur in other immune cells involved in AA pathogenesis, such as CD4⁺ and CD8⁺ T cells, thereby cooperatively contributing to disease progression.

Although CD8⁺ T cells are considered the primary pathogenic effectors in AA, NK cells contribute to disease development by regulating the activation and proliferation of CD8⁺ T cells^11^, ^48^. Our findings suggest that Ash1L deficiency-induced NK cell dysfunction amplifies inflammatory responses and exacerbates CD8⁺ T cell-mediated hematopoietic damage, providing new insights into the immune microenvironmental mechanisms of AA. Future *in vivo* co-transplantation models will be valuable to further elucidate the crosstalk between NK cells and CD8⁺ T cells. Notably, Ash1L is also essential for the self-renewal and homeostasis maintenance of hematopoietic stem and progenitor cells (HSPCs)^49^. Therefore, dysregulated Ash1L expression in both immune cells and HSPCs may synergistically contribute to bone marrow failure in AA. Comprehensive elucidation of Ash1L function across different cell types will deepen our understanding of AA pathogenesis and clonal evolution.

Cells continually encounter endogenous and exogenous genotoxic stressors, triggering DDR^39^. Single-cell transcriptomic studies have revealed aberrant splicing and polyadenylation biases in DDR-related transcripts in residual AA HSPCs. These findings indicate genomic instability in these cells. This genomic instability may contribute to the late progression of AA to malignant hematologic disorders such as myelodysplastic syndrome or leukemia^50^. In the present study, we demonstrate that downregulated Ash1L expression in AA leads to chromatin compaction, diminished DNA repair capacity, and persistent genomic instability in NK cells. These findings suggest that, compared with healthy individuals, patients with AA may be more susceptible to DNA repair defects. This susceptibility occurs when they are exposed to increasingly prevalent environmental stressors in modern life, such as physical or ionizing radiation, pharmacological agents, toxins, and viral or bacterial infections. Such exposure may thereby exacerbate immune dysfunction and hematopoietic failure.

Previous studies have shown that Ash1L participates in ultraviolet-induced NER through the Ash1L-MRG15 complex^51^. Considering the extensive crosstalk among different DNA repair pathways^38^, although our study primarily confirms the impact of Ash1L on HR and NHEJ repair in AA NK cells, its potential regulatory roles in other repair pathways cannot be excluded. Furthermore, silver-stained mass spectrometry results indicate that, under DNA damage conditions, Ash1L may interact with multiple proteins associated with genomic stability such as RBBP4 and RBBP7, suggesting that it could coordinately participate in DDR through multi-protein complexes. It should be noted that although the Ash1L-C fragment used in our IP-MS analysis retains core methyltransferase activity, it may not fully recapitulate the interaction network of the full-length protein. Future studies could employ full-length Ash1L for a more comprehensive interactome analysis. Further elucidation of these mechanisms will be essential for a comprehensive understanding of the multifaceted role of Ash1L in maintaining genomic stability in immune cells and in the pathogenesis of AA.

The cGAS-STING pathway senses cytosolic DNA, activating type I interferons and proinflammatory cytokines to reshape the immune microenvironment^52^, ^53^. Inhibition of cGAS has been shown to enhance HR repair and reduce genomic instability^54^. Here, Ash1L deficiency drives STING activation in NK cells, contributing to AA's inflammatory microenvironment. Given the multifaceted roles of Ash1L in chromatin remodeling, direct manipulation of its expression may carry potential risks; thus, targeting downstream STING signaling appears more feasible. Several STING inhibitors, including H-151, have demonstrated therapeutic efficacy in models of autoimmune and neurodegenerative diseases^55^, ^56^. Andro, a clinically approved natural compound, inhibits cGAS-STING and NF-κB, enhances DDR, and reduces proinflammatory cytokines. Compared with classical STING inhibitors such as H-151, Andro exhibits distinct advantages and limitations. H-151 directly targets the Cys91 residue of STING via covalent modification to block STING palmitoylation and activation, conferring high target specificity^57^. However, its clinical application is still in the early stage, with safety and efficacy yet to be fully established. In contrast, as a natural compound, Andro has favorable safety and tolerability and has been widely used clinically. Although Andro may inhibit the STING pathway indirectly through antioxidant and anti-inflammatory effects, its multi-target actions (including suppression of NF-κB and PI3K/AKT pathways) may confer greater advantages in the complex immune microenvironments of AA. Future studies are warranted to clarify the efficacy differences between Andro and specific STING inhibitors.

Unlike conventional immunosuppressive agents that primarily exert broad immune suppression, Andro acts through multi-target mechanisms with favorable safety and tolerability profiles. Therefore, Andro holds considerable promise for clinical translation in AA and other immune-dysregulated BMF diseases. Although Andro demonstrated therapeutic efficacy in AA animal models, there is currently no direct evidence that it can replace standard IST regimens. Given its previously reported immunoregulatory properties and the findings of this study showing that Andro inhibits STING signaling and restores NK cell function in both cellular and murine models, we propose that Andro is more likely to serve as an adjunctive therapy to IST, aiming to enhance therapeutic efficacy and reduce adverse effects. The multi-target actions and favorable safety profile of Andro represent its key advantages; however, its clinical utility requires further validation through future clinical trials. Limitations of this study include the evaluation of only Ash1L mRNA expression in clinical samples, without protein-level validation. This was primarily due to the lack of a validated Ash1L-specific antibody suitable for flow cytometry and the extremely limited NK cell yields from AA patients owing to pancytopenia. Additionally, most functional experiments were performed using NK cell lines, with only limited validation in primary NK cells. Future studies utilizing advanced approaches, such as single-cell proteomics and CRISPR-Cas9 mediated gene editing, will be essential to further validate our findings in primary NK cells and *in vivo* models.

In summary, this study identifies Ash1L as a key epigenetic regulator essential for maintaining DDR and genomic stability in NK cells. Ash1L deficiency results in persistent DNA damage, aberrant activation of the cGAS-STING pathway, excessive inflammatory responses, and exacerbation of BMF. Andro enhances DNA repair capacity and suppresses STING signaling, thereby representing a potential therapeutic strategy that integrates epigenetic regulation with anti-inflammatory effects for the treatment of AA and related immune-mediated BMF diseases.

## Supplementary Material

Supplementary figures, tables, and files.

## Data Availability

All other relevant data are available from the corresponding author upon reasonable request.
