# Supplementary material for: Targeting Ash1L-STING Axis Restores NK Cell Function and Ameliorates Immune-Mediated Bone Marrow Failure Diseases
Source: Int J Biol Sci. 2026 Jul 13;22(12):6653–69. doi: 10.7150/ijbs.131763 (PMC13412093; doi:10.7150/ijbs.131763)
Supplement: Supplementary file 1 — Supplementary figures, tables, and files. [file ijbsv22p6653s1.pdf]

Supplementary Figure legends

Supplementary Figure 1. Clinical correlation and phenotypic verification of Ash1L knockdown in NK cell lines

(A) Additional correlation analyses between Ash1L expression and blood parameters in AA and AA-PR patients. (B) Serum cytokine levels in AA, AA-PR, AA-CR, and HC groups. (C) Additional correlation analyses between Ash1L expression and cytokine levels. (D) Ash1L knockdown efficiency in YT cells by Western blot and RT-qPCR. (E) Viability of control and Ash1L-knockdown YT cells (CCK-8). (F) Apoptosis in control and Ash1L-knockdown YT cells. (G) Cell cycle distribution in control and Ash1L-knockdown YT cells. (H) qPCR analysis of gene expression following Ash1L knockdown in NK-92 cells. *$p < 0.05$, **$p < 0.01$, ***$p < 0.001$, one-way ANOVA.

Supplementary Figure 2. Ash1L deficiency aggravates chemotherapy- and irradiation-induced DNA damage

(A) Nucleosome digestion in control and Ash1L-knockdown YT cells. (B) γH2AX levels in NK-92 cells treated with escalating CDDP concentrations. (C) Time-course of γH2AX induction by 40 μM CDDP in NK-92 and U2OS cells. (D) γH2AX levels following X-ray irradiation (4 Gy). (E) Immunofluorescence of γH2AX foci with or without X-ray irradiation in U2OS cells. Scale bar, 25 μm. (F-G) Quantification of γH2AX IF and comet assay results. Data are shown as mean ± SD. (H) Comet assay evaluating DNA damage in control and Ash1L-knockdown U2OS cells. Scale bar, 100 μm. *$p < 0.05$, **$p < 0.01$, ns, not significant, one-way ANOVA.

Supplementary Figure 3. Ash1L regulates DNA damage repair factor recruitment

(A–B) Quantification of fluorescence intensity in Figure 3A and 3B. (C) IF analysis of 53BP1, BRCA1, and γH2AX following Ash1L knockdown in U2OS cells. Scale bar, 25 μm. Data are shown as mean ± SD; *$p < 0.05$, **$p < 0.01$, one-way ANOVA.

Supplementary Figure 4. Toxicity evaluation and molecular effect of Andrographolide

(A) Micronuclei formation in U2OS cells after Ash1L knockdown. Scale bar, 5 µm. (B) Reactive oxygen species (ROS) levels following Ash1L knockdown in U2OS cells. Scale bar, 100 µm. (C) Dose-response curve showing the inhibitory effect of Andro on NK-92 cells and the corresponding IC_{50} value (17.06 µM). (D) Western blot and RT-qPCR analysis of Ash1L protein and mRNA expression levels in NK-92 cells following treatment with various concentrations of Andro. $^{*}p < 0.05$, $^{**}p < 0.01$, ns, not significant, one-way ANOVA.

sFigure 1

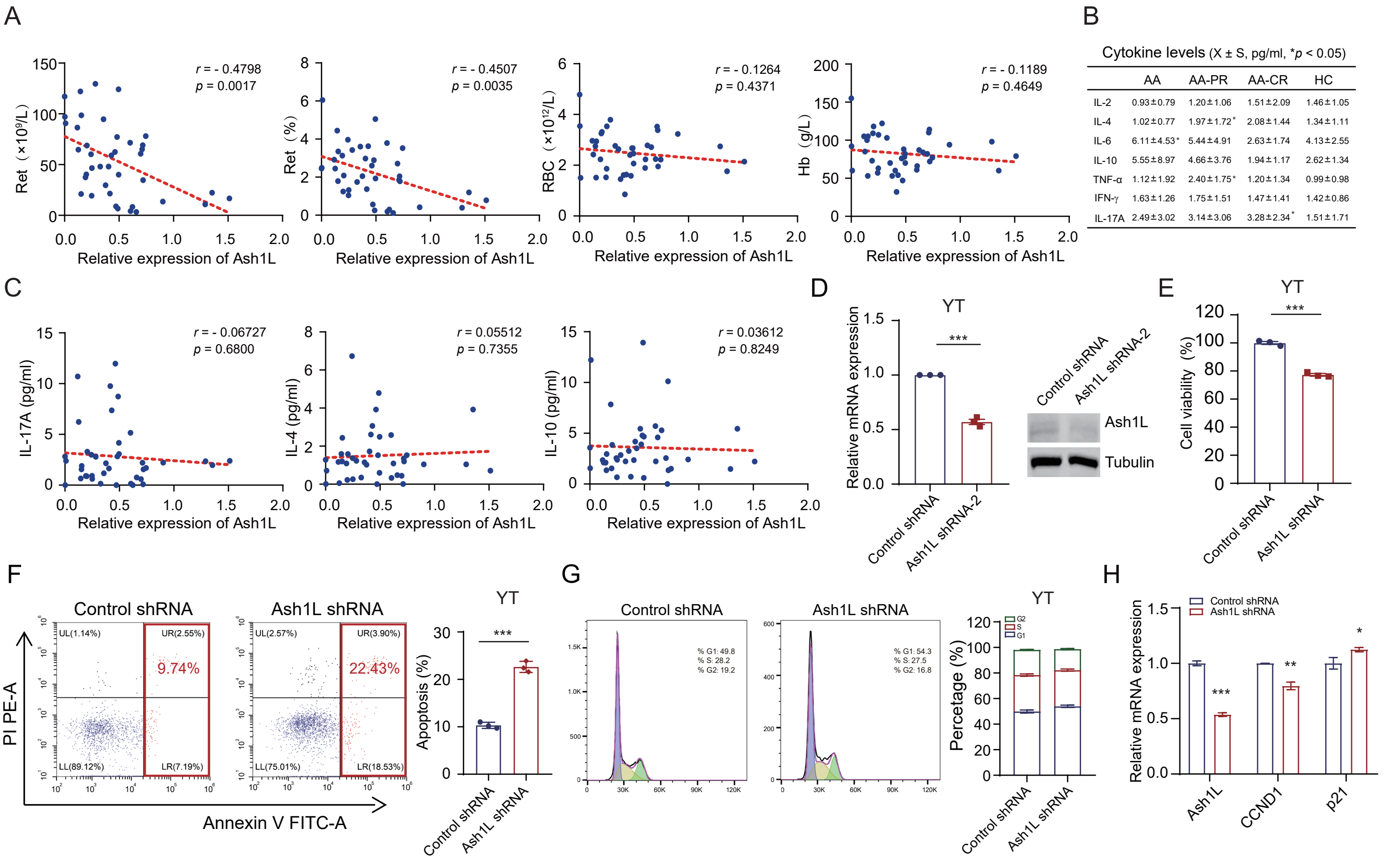

B

Cytokine levels (X ± S, pg/ml, *$p < 0.05$)

	AA	AA-PR	AA-CR	HC
IL-2	0.93±0.79	1.20±1.06	1.51±2.09	1.46±1.05
IL-4	1.02±0.77	1.97±1.72*	2.08±1.44	1.34±1.11
IL-6	6.11±4.53*	5.44±4.91	2.63±1.74	4.13±2.55
IL-10	5.55±8.97	4.66±3.76	1.94±1.17	2.62±1.34
TNF-α	1.12±1.92	2.40±1.75*	1.20±1.34	0.99±0.98
IFN-γ	1.63±1.26	1.75±1.51	1.47±1.41	1.42±0.86
IL-17A	2.49±3.02	3.14±3.06	3.28±2.34*	1.51±1.71

sFigure 2

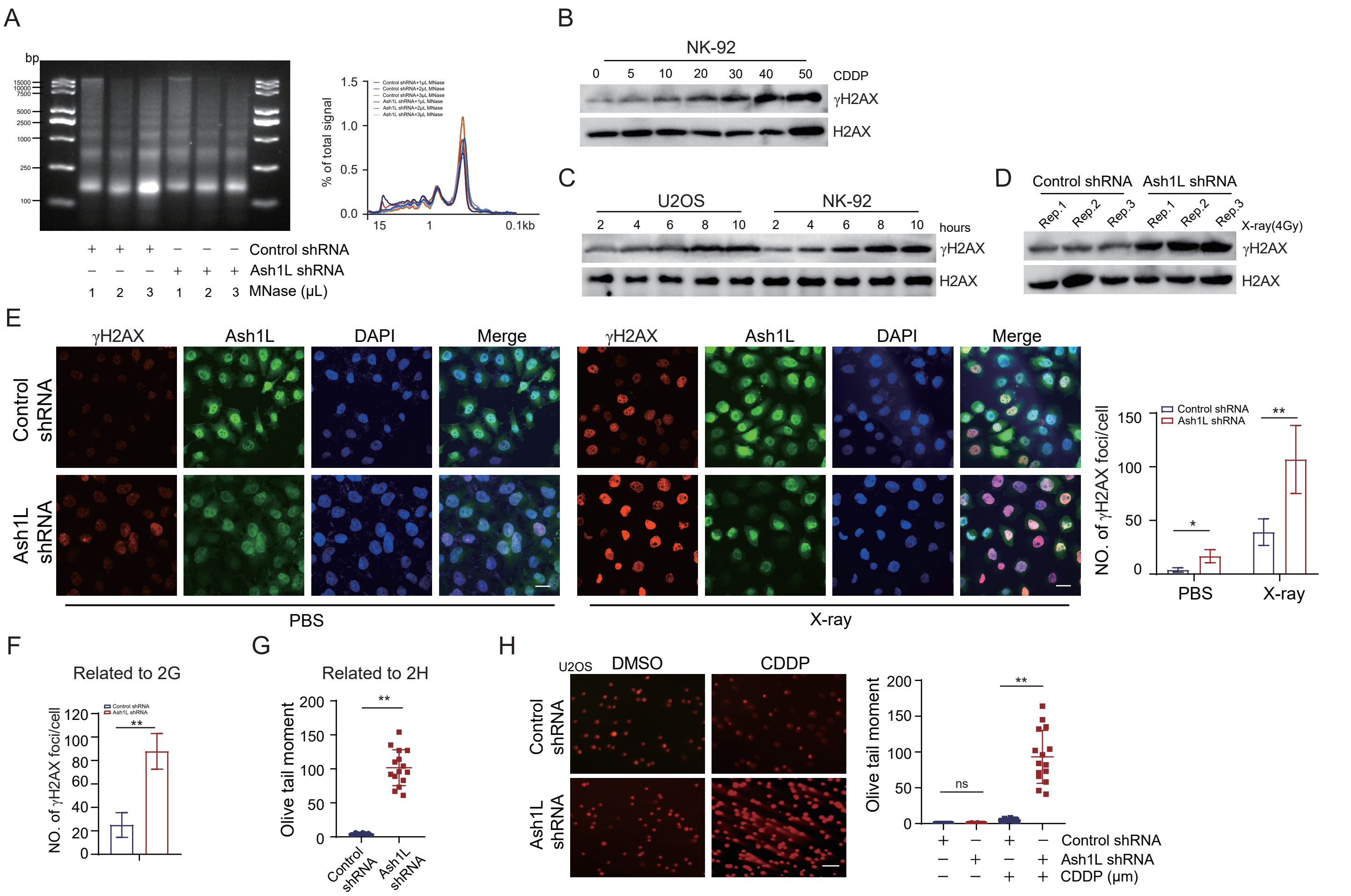
A
bp
Control shRNA
Ash1L shRNA
MNase (μL)
% of total signal
0.1kb
B
NK-92
CDDP
γH2AX
H2AX
C
U2OS
NK-92
hours
γH2AX
H2AX
D
Control shRNA
Ash1L shRNA
Rep.1
Rep.2
Rep.3
X-ray(4Gy)
γH2AX
H2AX
E
γH2AX
Ash1L
DAPI
Merge
Control shRNA
Ash1L shRNA
PBS
X-ray
NO. of γH2AX foci/cell
F
Related to 2G
NO. of γH2AX foci/cell
G
Related to 2H
Olive tail moment
Control shRNA
Ash1L shRNA
H
U2OS
DMSO
CDDP
Control shRNA
Ash1L shRNA
Olive tail moment
ns
Control shRNA
Ash1L shRNA
CDDP (μm)

sFigure 3

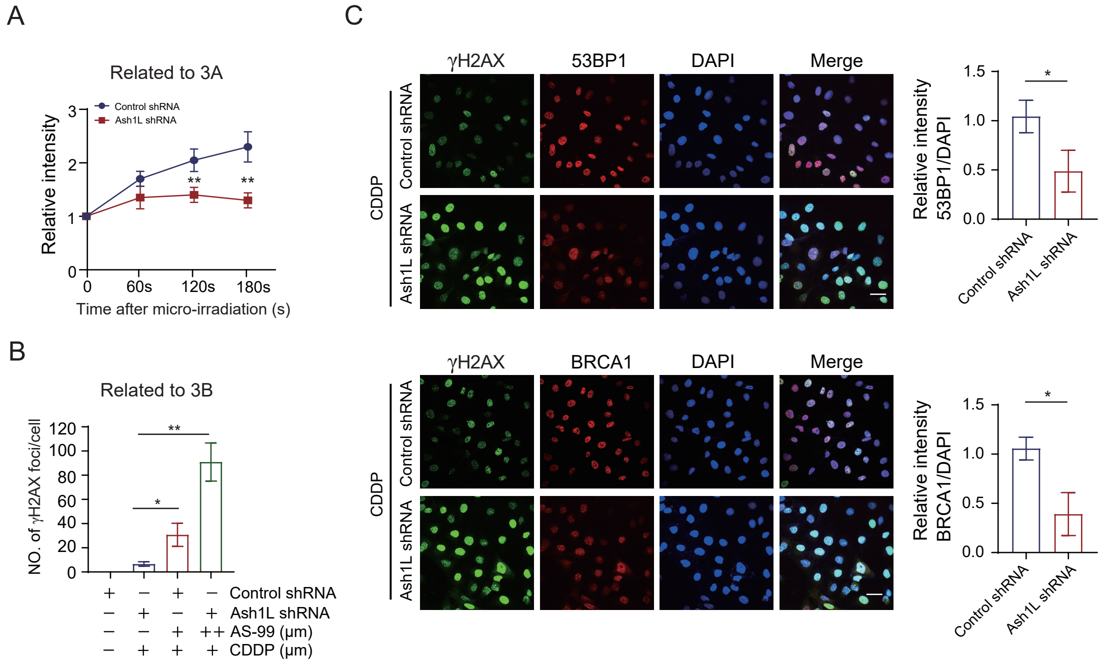
A
Related to 3A
Control shRNA
Ash1L shRNA
Relative intensity
Time after micro-irradiation (s)
0
60s
120s
180s
B
Related to 3B
NO. of γH2AX foci/cell
Control shRNA
Ash1L shRNA
AS-99 (μm)
CDDP (μm)
C
γH2AX
53BP1
DAPI
Merge
CDDP
Control shRNA
Ash1L shRNA
Relative intensity 53BP1/DAPI
γH2AX
BRCA1
DAPI
Merge
Relative intensity BRCA1/DAPI

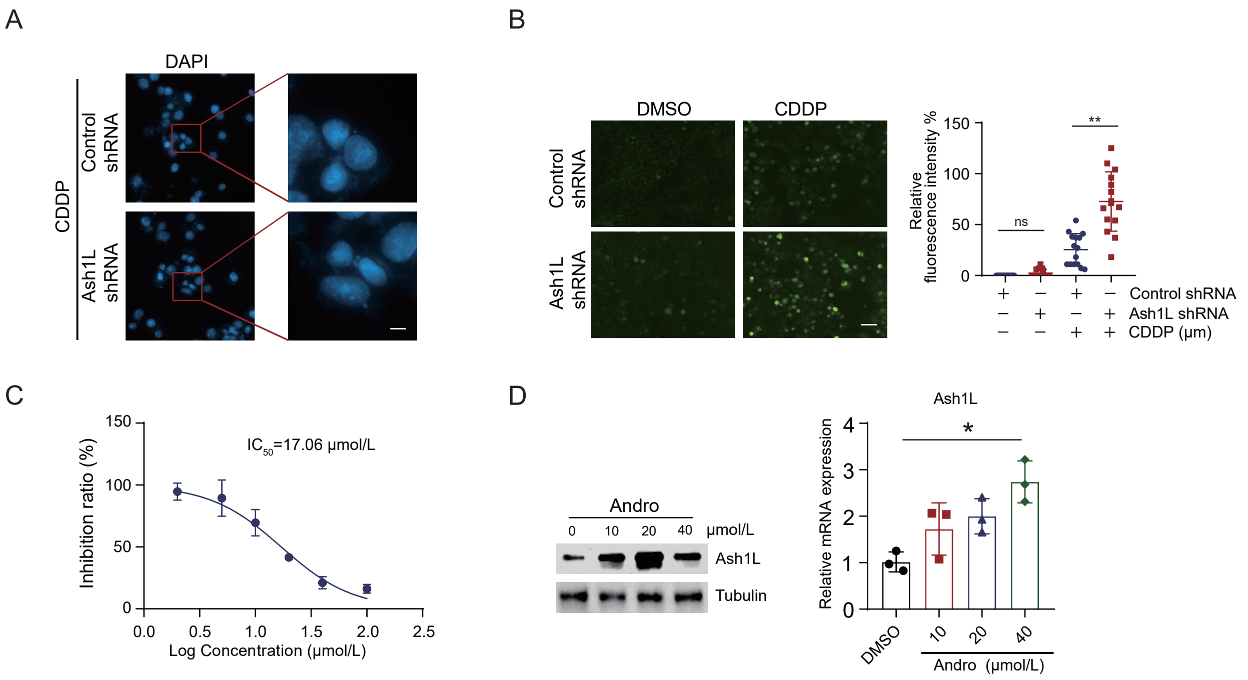
A
DAPI
CDDP
Control shRNA
Ash1L shRNA
B
DMSO
CDDP
Control shRNA
Ash1L shRNA
Relative fluorescence intensity %
150
100
50
0
ns
**
+ − + − Control shRNA
− + − + Ash1L shRNA
− − + + CDDP (μm)
C
IC50=17.06 μmol/L
Inhibition ratio (%)
150
100
50
0
0.0 0.5 1.0 1.5 2.0 2.5
Log Concentration (μmol/L)
D
Andro
0 10 20 40 μmol/L
Ash1L
Tubulin
Ash1L
Relative mRNA expression
4
3
2
1
0
*
DMSO
10
20
40
Andro (μmol/L)

Supplementary File 1

Patient Demographics and Clinical Characteristics

	AA	AA-CR	AA-PR	Healthy control
Age (years)	41.8 （7-78）	41.65 （9-60）	43.25 （21-47）	42.43 （17-67）
Male/Female	8/12	8/9	9/11	14/16
WBC（$\times 10^9$/L）	2.819±1.233	5.755±2.648	3.873±2.176	5.367±2.461
RBC（$\times 10^{12}$/L）	2.828±0.876	3.380±0.772	2.09±0.529	3.669±0.804
Hb（g/L）	93.26±25.96	111.5±25.51	70.16±16.91	96.54±33.39
PLT（$\times 10^9$/L）	35.11±29.48	117.6±77.08	34.37±25.75	225.9±109.2
Ret（%）	2.12±1.20	2.13±1.02	3.59±3.15	2.31±1.53
Ret（$\times 10^9$/L）	61.48±39.37	72.55±34.49	75.51±71.39	78.50±45.97

Supplementary File 2

Lentiviral shRNA sequences

shRNAs	Sequences
**Control**	CCGGTTCTCCGAACGUGTCACGTCTCGAGACGTGACACGTTCGGAGAATTTTTG
**shAsh1L-1**	CCGGTGCTGTTGGAGAGCGATATAACTCGAGTTATATCGCTCTCCAACAGCA TTTTTG
**shAsh1L-2**	CCGGGAGTCGATTGATCCAATTAAACTCGAGTTTAATTGGATCAATCGACTC TTTTTG

Note: Red color indicates the targeting sequence against the corresponding genes.

qRT-PCR primers

Gene	Primer（5'-3'）	
Ash1L	Forward	ACACTGTCCTTCAAAACGAGAC
	Reverse	GAAGAGTAGATGGCGTTGCATTA
IFN-β	Forward	GCTTGGATTCCTACAAAGAAGCA
	Reverse	ATAGATGGTCAATGCGGCGTC
IL-18	Forward	TCTTCATTGACCAAGGAAATCGG
	Reverse	TCCGGGGTGCATTATCTCTAC
IL-6	Forward	ACTCACCTCTTCAGAACGAATTG
	Reverse	CCATCTTTGGAAGGTTCAGGTTG
IL-1β	Forward	AGCTACGAATCTCCGACCAC
	Reverse	CGTTATCCCATGTGTCGAAGAA
CXCL9	Forward	CCAGTAGTGAGAAAGGGTCGC
	Reverse	AGGGCTTGGGGCAAATTGTT
CXCL10	Forward	GTGGCATTCAAGGAGTACCTC
	Reverse	TGATGGCCTTCGATTCTGGATT
CCND1	Forward	GCTGCGAAGTGGAAACCATC
	Reverse	CCTCCTTCTGCACACATTTGAA
P21	Forward	TGTCCGTCAGAACCCATGC
	Reverse	AAAGTCGAAGTTCCATCGCTC
β-actin	Forward	CATGTACGTTGCTATCCAGGC
	Reverse	CTCCTTAATGTCACGCACGAT

Supplementary File 3

Mass Spectrometry Analysis of PKM2-containing Protein Complex - PeptideGroups（Proteins with unique peptides ≥5, coverage ≥15%）

Protein	Protein Name	Gene Name	Proteins	Peptides	Unique Peptides	Coverage	MolWeight	Intensity Cis-OE	Intensity OE
A0A7I2YQU9	ASH1 like histone lysine methyltransferase	ASH1L	7	63	63	21.2	325.52	240160000	242090000
P52732	Kinesin-like protein KIF11	KIF11	6	41	41	45.4	119.16	15639000	9451200
P15924	Desmoplakin	DSP	8	31	31	15.7	331.77	3352600	5120700
A0A8I5KWT8	Myosin heavy chain 9	MYH9	20	30	27	20.5	229.18	2398600	3215900
Q00839	Heterogeneous nuclear ribonucleoprotein U	HNRNPU	22	16	16	25.7	90.583	5462300	4733300
B3KWX6	cDNA FLJ44127 fis, clone THYMU2006420, highly similar to Mortality factor 4-like protein 2		8	19	16	68.9	30.45	98889000	65960000
Q5T749	Keratinocyte proline-rich protein	KPRP	1	16	16	47.8	64.135	6556800	3475900
P36578	Large ribosomal subunit protein uL4	RPL4	10	15	15	38.9	47.697	5715500	4498300
B2RDD7	Protein arginine N-methyltransferase 5		16	14	14	32.7	72.709	3926200	3955400
A5D8W6	Mortality factor 4 like 1	MORF4L1	12	17	14	59.4	37.26	19734000	12554000
B3KP90	protein-serine/threonine phosphatase		14	13	13	38.8	52.709	5884500	3016200
Q09028	Histone-binding protein RBBP4	RBBP4	10	22	13	72.7	47.655	112080000	60266000
P11142	Heat shock cognate 71 kDa protein	HSPA8	28	14	12	37	70.897	1994200	1488300
P39023	Large ribosomal subunit protein uL3	RPL3	15	12	12	39.7	46.108	4479800	4775600
Q02413	Desmoglein-1	DSG1	1	12	12	15.3	113.75	1970900	2287500
A0A7P0TB36	78 kDa glucose-regulated protein	HSPA5	5	12	11	22.3	69.01	1034100	982560
P23396	Small ribosomal subunit protein uS3	RPS3	16	11	11	57.2	26.688	3345400	2574200
P05388	Large ribosomal subunit protein uL10	RPLP0	19	10	10	45.4	34.273	4339000	2885700
P15880	Small ribosomal subunit protein uS5	RPS2	18	10	10	39.6	31.324	6279100	5035500
A0A087X0X3	Heterogeneous nuclear ribonucleoprotein M	HNRNPM	14	10	10	20.4	77.569	555390	479130
P62424	Large ribosomal subunit protein eL8	RPL7A	4	10	10	31.6	29.995	6343700	4391400
Q6FHQ0	RBBP7 protein (Fragment)	RBBP7	8	17	10	53.4	47.82	6382700	2590200
V9HVZ4	Glyceraldehyde-3-phosphate dehydrogenase	HEL-S-162eP	8	9	9	48.7	36.053	1834900	2488800
Q0QEN7	ATP synthase subunit beta (Fragment)	ATP5B	7	9	9	34.4	48.113	237240	270340
P18124	Large ribosomal subunit protein uL30	RPL7	5	9	9	41.5	29.225	8323900	6461300
A0A140VK00	Testicular tissue protein Li 227		5	9	9	38.9	34.258	1471000	1160200
Q96IR1	RPS4X protein (Fragment)	RPS4X	11	9	9	40.7	27.259	4494700	2394400
P62906	Large ribosomal subunit protein uL1	RPL10A	2	9	9	37.3	24.831	6120000	5584500
Q8TBK5	60S ribosomal protein L6	RPL6	12	9	9	35.8	32.741	3830800	2403800
Q15208	Serine/threonine-protein kinase 38	STK38	8	11	9	36.6	54.19	1436000	666530
A0A384P5Q0	Catalase		5	8	8	20.7	59.755	530870	405150
A0A0S2Z487	Junction plakoglobin isoform 1 (Fragment)	JUP	9	14	8	27.7	81.744	1196800	1346800
B3KM80	Nucleolin	NCL	15	8	8	20.3	58.554	1710400	1264100
P46781	Small ribosomal subunit protein uS4	RPS9	7	8	8	36.1	22.591	4411300	3283000
Q6NXR8	Small ribosomal subunit protein eS1	RPS3A	16	8	8	36.4	29.975	4950300	3028700
Q5JR94	40S ribosomal protein S8	RPS8	4	8	8	45.7	24.205	6282300	5699100
F4ZW64	NF90a		15	8	8	17.4	75.96	803860	636540
Q9NZT1	Calmodulin-like protein 5	CALML5	2	8	8	78.1	15.892	1808100	3086800
E2DRY6	phosphopyruvate hydratase		13	7	7	32	36.554	349520	1307900
H0YMW4	Annexin	ANXA2	27	7	7	24.8	41.899	784190	1003800
P13639	Elongation factor 2	EEF2	7	7	7	17.8	95.337	169300	491440
F8W1R7	Myosin light chain 6	MYL6	17	7	7	51.7	16.29	2993800	1450500
P62249	Small ribosomal subunit protein uS9	RPS16	7	7	7	41.1	16.445	3804500	3003000
P62280	Small ribosomal subunit protein uS17	RPS11	4	7	7	44.9	18.431	2404900	2118200

P68871	Hemoglobin subunit beta	HBB	67	8	7	65.3	15.998	2869800	2543500
B7Z4R3	T-complex protein 1 subunit beta		6	7	7	27.8	50.943	140740	397470
B4DY09	Interleukin enhancer binding factor 2	ILF2	7	7	7	31.8	38.91	706410	640710
P12273	Prolactin-inducible protein	PIP	1	6	6	43.8	16.572	1283800	1094500
A0A087WXM6	Large ribosomal subunit protein uL22 (Fragment)	RPL17	14	6	6	45	19.586	3003500	2021300
A0A7I2V4N0	Heterogeneous nuclear ribonucleoproteins A2/B1	HNRNPA2B1	13	6	6	48.1	18.051	494860	629550
P30050	Large ribosomal subunit protein uL11	RPL12	3	6	6	54.5	17.818	1341100	1130800
B4DXZ6	FMR1 autosomal homolog 1	FXR1	14	6	6	17.1	68.326	217510	98364
Q8WXX5	DnaJ homolog subfamily C member 9	DNAJC9	2	6	6	33.1	29.909	679910	267000
Q96P63	Serpin B12	SERPINB12	2	6	6	19.5	46.276	542800	843510
Q8N254	ATP-dependent RNA helicase		5	6	6	20.8	46.643	233270	136610
P05141	ADP/ATP translocase 2	SLC25A5	12	5	5	19.5	32.852	859610	790490
P05387	Large ribosomal subunit protein P2	RPLP2	3	6	5	84.3	11.665	2497200	1142900
B2R4M6	Protein S100		2	5	5	56.1	13.21	1060300	739930
A0A7I2YQC0	Nucleophosmin	NPM1	18	5	5	26.3	35.713	1088700	971770
B4DJ51	Calmodulin 1 (Phosphorylase kinase, delta), isoform CRA_a	HEL-S-72	14	5	5	45.6	16.837	5199100	4064500
P10809	60 kDa heat shock protein, mitochondrial	HSPD1	24	5	5	15.4	61.054	223700	627900
B2RDN9	cDNA, FLJ96699, highly similar to Homo sapiens thyroid autoantigen 70kDa (Ku antigen) (G22P1		6	5	5	16.1	69.784	131070	244060
P16403	Histone H1.2	H1-2	10	5	5	19.2	21.364	7400700	4185500
B2CIS9	Caspase 14, apoptosis-related cysteine peptidase	CASP14	3	5	5	27.7	27.679	663390	828710
Q5QTS3	Large ribosomal subunit protein uL13		14	5	5	26.6	23.662	2816700	2281200
Q7Z759	T-complex protein 1 subunit theta	CCT8	5	5	5	16.9	54.106	125750	104380
P52597	Heterogeneous nuclear ribonucleoprotein F	HNRNPF	4	6	5	22.9	45.671	1222400	549890
A0A2R8YEM3	Ribosomal protein L15 (Fragment)	RPL15	8	5	5	32.5	19.968	1812400	1500600
Q5T6W2	Heterogeneous nuclear ribonucleoprotein K	HNRNPK	14	5	5	20.8	41.807	433550	407540
A0A2R8Y811	Ribosomal protein S14 (Fragment)	RPS14	4	5	5	36.7	16.159	2490000	1696600
P62269	Small ribosomal subunit protein uS13	RPS18	4	5	5	32.2	17.718	2603500	1653000
P62851	Small ribosomal subunit protein eS25	RPS25	1	5	5	29.6	13.742	3184500	2006200
E9PKZ0	Large ribosomal subunit protein uL2 (Fragment)	RPL8	6	5	5	41.5	22.389	2874100	2484800
P62979	Ubiquitin-ribosomal protein eS31 fusion protein	RPS27A	42	5	5	42.3	17.965	11060000	8368500
M0R3D6	Large ribosomal subunit protein eL20 (Fragment)	RPL18A	12	5	5	37.6	16.714	2541300	2081800
Q13268	Dehydrogenase/reductase SDR family member 2, mitochondrial	DHRS2	5	5	5	22.1	29.926	1140000	840820
A8K7N0	Large ribosomal subunit protein eL14		7	5	5	28	23.646	3766500	1829200

Supplementary File 3

Mass Spectrometry Analysis of Ash1L-containing Protein Complex - PeptideGroups

Sequence	Missed cleavages	Mass	Proteins	Unique (Groups)	Charges	PEP	Score	Intensity	Intensity Cis-OE	Intensity OE
ADKEAAFDDAVEER	1	1564.7005	sp|Q09028|RBBP4	yes	2	1.4454E-08	151.08	14911000	9637200	5274100
ADKEAAFDDAVEERVINEEYK	2	2440.1394	sp|Q09028|RBBP4	yes	3	0.0092244	13.525	16173	16173	0
DEIFQVQWSPHNETILASSGTDR	0	2629.2409	sp|Q09028|RBBP4	yes	3	1.0221E-31	115.78	582450	365760	216690
EAAFDDAVEER	0	1250.5415	sp|Q09028|RBBP4	yes	2	4.62E-37	213.45	3061600	1849500	1212100
EGYGLSWNPNLSGHLLSASDDHTICLWDISAVPK	0	3751.7941	sp|Q09028|RBBP4	yes	4	1.2428E-81	129.66	799240	549420	249810
HPSKPDPSGECNPDLR	1	1804.8162	sp|Q09028|RBBP4	yes	2;3	5.8039E-32	193.62	807720	729350	78370
IGEEQSPEDAEDGPPELLFIHGGHTAK	0	2872.3515	sp|Q09028|RBBP4	yes	3;4	3.4496E-69	206.61	5488600	4185600	1302900
INHEGEVNR	0	1066.5156	sp|Q09028|RBBP4	no	2	6.3444E-10	163.2	1403600	29325	1374300
LHSFESHK	0	983.48248	sp|Q09028|RBBP4	yes	2	0.028926	78.178	161090	161090	0
LNVWDLSK	0	973.52328	sp|Q09028|RBBP4	no	2	0.00040881	176.75	7177000	4177200	2999800
SNNTSKPSHSVDAHTAEVNCLSFNPYSEFILATGSADK	1	4094.8916	sp|Q09028|RBBP4	yes	4	5.0433E-20	48.049	325830	278990	46836
TIFTGHTAVVEDVSWHLLHESLFGSVADDQK	0	3437.6892	sp|Q09028|RBBP4	yes	4	1.0622E-18	71.299	1006100	535750	470320
TPSSDVLVFDYTKHPSKPDPSGECNPDLR	2	3257.5299	sp|Q09028|RBBP4	yes	3;4	2.474E-33	89.913	1202800	888320	314530
YMPQNPCIIATK	0	1434.6999	sp|Q09028|RBBP4	yes	2	2.3785E-16	159.93	21735000	16005000	5730000
ASKEMFEDTVEER	1	1569.6981	sp|Q16576|RBBP7	yes	2	0.0050717	46.768	243820	181070	62747
DYALHWLVLGTHTSDEQNHLVVAR	0	2773.3936	sp|Q16576|RBBP7	yes	3;4	5.4278E-68	124.77	806850	546840	260010
EMFEDTVEER	0	1283.534	sp|Q16576|RBBP7	yes	2	0.00087043	108.3	1851000	1273100	577860
GEFGGFGSVTGK	0	1141.5404	sp|Q16576|RBBP7	yes	2	1.834E-10	163.43	1975600	1365400	610210
IGEEQSAEDAEDGPPELLFIHGGHTAK	0	2846.3359	sp|Q16576|RBBP7	yes	3;4	2.0707E-29	115.01	687380	448950	238420
LHTFESHKDEIFQVHWSPHNETILASSGTDR	1	3617.7288	sp|Q16576|RBBP7	yes	4	2.4417E-45	66.215	482330	406440	75897
PSHLVDAHTAEVNCLSFNPYSEFILATGSADK	0	3489.6511	sp|Q16576|RBBP7	yes	4	4.0044E-24	77.122	251150	212960	38197
VHIPNDDAQFDASHCDSDK	0	2169.9022	sp|Q16576|RBBP7	yes	3	1.8663E-19	107.46	935670	660190	275490
VHIPNDDAQFDASHCDSDKGEFGGFGSVTGK	1	3293.432	sp|Q16576|RBBP7	yes	4	8.8379E-08	36.377	630790	462160	168630
YMPQNPHIIATK	0	1411.7282	sp|Q16576|RBBP7	yes	2;3	0.0047308	92.445	886730	664220	222510
AGQFIIEYLGEVVSEQEFR	0	2213.1004	sp|Q9NR48|ASH1L	yes	2;3	4.1868E-287	330.54	13626000	7280000	6345800
CICGLYK	0	912.41974	sp|Q9NR48|ASH1L	yes	2	0.032446	71.575	5991900	3155400	2836500
CICGLYKDEGLMIQCDK	1	2101.9305	sp|Q9NR48|ASH1L	yes	3	3.8413E-24	146.37	1487000	880810	606220
CMVWQHCDCMGVNSDVEHYLCEQCDPR	0	3484.328	sp|Q9NR48|ASH1L	yes	4	3.8894E-36	129.44	1195600	751100	444510
CMVWQHCDCMGVNSDVEHYLCEQCDPRPVDR	1	3951.5772	sp|Q9NR48|ASH1L	yes	4	1.1163E-08	53.872	773910	503270	270640
DDGNIKSDVFMTQFSALQTAR	1	2343.1165	sp|Q9NR48|ASH1L	yes	3	0.0012353	84.266	262110	119280	142830
DEGLMIQCDK	0	1207.5213	sp|Q9NR48|ASH1L	yes	2	2.7746E-42	222.07	9328700	4742800	4585900
DFSPHYVPDNYK	0	1480.6623	sp|Q9NR48|ASH1L	yes	2;3	1.701E-67	228.54	2766800	1384200	1382500
DFSPHYVPDNYKR	1	1636.7634	sp|Q9NR48|ASH1L	yes	3	6.5478E-13	191.55	1330100	653850	676200
DKLDIFR	1	905.49707	sp|Q9NR48|ASH1L	yes	2	3.195E-17	169.97	18146000	9020900	9125600
DLGQEDDALPLIEEVLASQEQAANEIPSLEEPER	0	3746.801	sp|Q9NR48|ASH1L	yes	3	1.5151E-94	155.5	859020	324200	534820
DMPAGTELTYDYNFHSFNVEK	0	2477.0845	sp|Q9NR48|ASH1L	yes	2;3	3.9761E-70	220.49	17048000	8673200	8374900
EICDGIISYK	0	1196.5747	sp|Q9NR48|ASH1L	yes	2	5.4742E-16	187.82	15467000	7060000	8407100
EICDGIISYKDSSR	1	1641.7668	sp|Q9NR48|ASH1L	yes	2;3	0.00040208	80.623	210700	112750	97957
EPLKAGQFIIEYLGEVVSEQEFR	1	2680.3748	sp|Q9NR48|ASH1L	yes	3	0.0041462	26.55	17660	5987.9	11672
EQDVYICDYR	0	1359.5765	sp|Q9NR48|ASH1L	yes	2	0.0010273	99.855	16603000	8503700	8098800
EQDVYICDYRLDK	1	1715.7825	sp|Q9NR48|ASH1L	yes	3	0.030238	38.1	199830	94720	105110

GCVDDCLNR	0	1107.4437	sp|Q9NR48|ASH1L	yes	2	7.5329E-23	178.87	4379500	2018200	2361300
GHLSEEPSENINTPTR	0	1779.8388	sp|Q9NR48|ASH1L	yes	2;3	7.9846E-67	236.23	11050000	3975400	7074900
HEASAQIDEIVGETASEADSSETSVSEK	0	2905.2949	sp|Q9NR48|ASH1L	yes	3	1.3381E-175	231.63	1966800	904980	1061800
HEWVQCLER	0	1255.5768	sp|Q9NR48|ASH1L	yes	2	2.825E-09	142.56	9809400	5178100	4631400
HHVFLVR	0	906.51881	sp|Q9NR48|ASH1L	yes	2	0.000086066	148.12	1161300	798550	362730
HTSDNIHSASLYTR	0	1600.7594	sp|Q9NR48|ASH1L	yes	2;3	7.3344E-76	233	12133000	10628000	1505100
IDFQLPYDILWQWK	0	1863.956	sp|Q9NR48|ASH1L	yes	2;3	5.1933E-231	349.05	2019500	1215900	803550
IQRHEWVQCLER	1	1652.8205	sp|Q9NR48|ASH1L	yes	3	0.00078446	121.96	275400	0	275400
ISDPLDLITIEK	0	1355.7548	sp|Q9NR48|ASH1L	yes	2	1.0334E-11	160.98	16608000	6389700	10218000
KDFSPHYVPDNYK	1	1608.7573	sp|Q9NR48|ASH1L	yes	2;3	8.1302E-34	248.59	12202000	6522100	5679700
KDFSPHYVPDNYKR	2	1764.8584	sp|Q9NR48|ASH1L	yes	3;4	0.00096773	72.846	371920	20436	351480
KGCVDDCLNR	1	1235.5387	sp|Q9NR48|ASH1L	yes	2	1.1429E-16	178.94	555080	392630	162460
KPDVPLYK	1	958.54877	sp|Q9NR48|ASH1L	yes	2	0.0022843	133.68	3718100	1845100	1873000
LAAAEENIEVAR	0	1284.6674	sp|Q9NR48|ASH1L	yes	2	1.5283E-33	179.95	24760000	12261000	12499000
LLSHINR	0	851.49774	sp|Q9NR48|ASH1L	yes	2	0.028733	92.319	47400	46892	508.19
LNQILLNLLEK	0	1309.7969	sp|Q9NR48|ASH1L	yes	2	1.2893E-59	217.12	20120000	10783000	9337300
MIEQYHNHSDHYCLNLDSGMVIDSYR	0	3196.3801	sp|Q9NR48|ASH1L	yes	3;4	8.7405E-69	206.61	7124000	3736100	3387900
MIFAECSPNTCPCGEQCCNQR	0	2617.9899	sp|Q9NR48|ASH1L	yes	2;3	1.3058E-113	223.26	7660000	3987600	3672400
NADYYEKISDPLDLITIEK	1	2239.126	sp|Q9NR48|ASH1L	yes	3	0.00027907	47.491	24381	0	24381
NAIDVTYLLEEGSGR	0	1635.8104	sp|Q9NR48|ASH1L	yes	2	5.3875E-106	241.56	25103000	12356000	12746000
NAIDVTYLLEEGSGRK	1	1763.9054	sp|Q9NR48|ASH1L	yes	3	0.012683	22.724	71642	25252	46390
NSQPMATHK	0	1012.476	sp|Q9NR48|ASH1L	yes	2	0.0010693	86.205	61602	61602	0
